# Normothermic machine perfusion versus static cold storage in donation after circulatory death kidney transplantation: a randomized controlled trial

**DOI:** 10.1038/s41591-023-02376-7

**Published:** 2023-05-25

**Authors:** Sarah A. Hosgood, Christopher J. Callaghan, Colin H. Wilson, Laura Smith, Joanne Mullings, Jennifer Mehew, Gabriel C. Oniscu, Benedict L. Phillips, Lucy Bates, Michael L. Nicholson

**Affiliations:** 1 Department of Surgery, University of Cambridge, Addenbrooke’s Hospital, Cambridge, UK; 2 Department of Nephrology and Transplantation, Guy’s Hospital, Guy’s and St. Thomas’ NHS Foundation Trust, London, UK; 3 Freeman Hospital, Institute of Transplantation, Newcastle upon Tyne Hospitals NHS Trust and Institute of Cellular Medicine, Newcastle University, Newcastle upon Tyne, UK; 4 NHS Blood and Transplant (NHSBT) Clinical Trials Unit, NHSBT Stoke Gifford, Bristol, UK; 5 Edinburgh Transplant Centre, Royal Infirmary of Edinburgh, Edinburgh, UK

**Keywords:** Randomized controlled trials, Surgery

## Abstract

Kidney transplantation is the optimal treatment for end-stage renal disease, but it is still severely limited by a lack of suitable organ donors. Kidneys from donation after circulatory death (DCD) donors have been used to increase transplant rates, but these organs are susceptible to cold ischemic injury in the storage period before transplantation, the clinical consequence of which is high rates of delayed graft function (DGF). Normothermic machine perfusion (NMP) is an emerging technique that circulates a warmed, oxygenated red-cell-based perfusate through the kidney to maintain near-physiological conditions. We conducted a randomized controlled trial to compare the outcome of DCD kidney transplants after conventional static cold storage (SCS) alone or SCS plus 1-h NMP. A total of 338 kidneys were randomly allocated to SCS (*n* = 168) or NMP (*n* = 170), and 277 kidneys were included in the final intention-to-treat analysis. The primary endpoint was DGF, defined as the requirement for dialysis in the first 7 d after transplant. The rate of DGF was 82 of 135 (60.7%) in NMP kidneys versus 83 of 142 (58.5%) in SCS kidneys (adjusted odds ratio (95% confidence interval) 1.13 (0.69–1.84); *P* = 0.624). NMP was not associated with any increase in transplant thrombosis, infectious complications or any other adverse events. A 1-h period of NMP at the end of SCS did not reduce the rate of DGF in DCD kidneys. NMP was demonstrated to be feasible, safe and suitable for clinical application. Trial registration number: ISRCTN15821205.

## Abstract (web summary)

In an open-label, randomized controlled trial, normothermic machine perfusion of kidneys from donation after circulatory death was found to be feasible and safe but did not reduce the rate of delayed graft function compared to static cold storage.

## Main

Kidney transplantation is one of the major successes of modern medicine in the 20th century, transforming and extending the lives of many thousands of patients. However, a longstanding limitation of kidney transplantation is that the supply of transplantable organs does not meet demand^1^. One response to this has been the increasing use of kidneys from donation after circulatory death (DCD) donors^2^.

For more than 50 years, the traditional method of pre-transplant kidney preservation has been static cold storage (SCS) in ice^3,4^. This involves flushing the kidney to remove donor blood, cooling with a preservation solution at 4 °C and storage on ice while arrangements are made for transplantation. SCS works by reducing the metabolic rate to around 5% of normal, but because this occurs in an anoxic environment, anerobic metabolism ensues^5^. This leads to the depletion of cellular energy in the form of adenosine triphosphate (ATP) and the accumulation of succinate, which drives the production of the reactive oxygen species that underlie ischemia reperfusion injury^6^. SCS is simple, effective and inexpensive and is still the standard of care in the United Kingdom (UK). However, prolonged cold ischemic injury can lead to acute tubular necrosis, the clinical consequence of which is delayed graft function (DGF), requiring a period of post-transplant dialysis support.

DCD takes place after cessation of cardiac activity in the donor, leading to an inevitable period of warm ischemia, which can cause acute kidney injury. In addition, DCD kidneys are more susceptible to cold storage injury^7^. These factors lead to higher rates of DCD grafts never functioning (primary non-function (PNF)) and higher rates of DGF. There is evidence that DGF increases the risk of acute rejection, prolongs hospital stay and could adversely affect long-term allograft survival rates^8,9^.

Normothermic machine perfusion (NMP) is an emerging technique that uses cardiopulmonary bypass technology with extracorporeal membrane oxygenation to perfuse kidneys with a warmed and oxygenated red-cell-based plasma-free solution^10^. This maintains an organ in a near-physiological state, restoring function ex vivo and, therefore, allowing functional testing. Experimental studies using animal models suggest that kidney NMP has a conditioning effect with maintenance of more stable acid–base homeostasis and a reduction in renal tubular injury, when compared to SCS^11^. NMP also enables a degree of metabolic resuscitation by replenishing ATP levels that have been depleted because of a combination of warm and cold ischemia^12^.

The first randomized controlled trial of NMP in liver transplantation demonstrated that, compared to conventional SCS, NMP reduced early allograft dysfunction. There was no effect on bile duct complications, graft survival and patient survival^13^. As a result, NMP is finding increasing clinical application in liver transplantation. The technology is rapidly developing as a method of assessment and treatment to increase organ utilization and has proved to be more cost-effective than SCS^14,15^. There is currently a paucity of evidence for NMP in kidney transplantation. Nonetheless, with high rates of kidney discard^16^, there is clear potential for the introduction of NMP technology.

Our non-randomized pilot study in kidney transplantation suggested that a 1-h period of NMP, delivered at the end of the cold storage period and immediately before transplantation, reduced the rate of DGF in extended criteria donor kidneys^17^. Here we report the first multicenter, randomized controlled trial comparing NMP with conventional SCS in DCD kidney transplantation. The primary endpoint was DGF, defined as the requirement for dialysis in the first 7 d after transplant.

## Results

### Patient population

**Fig. 1:**
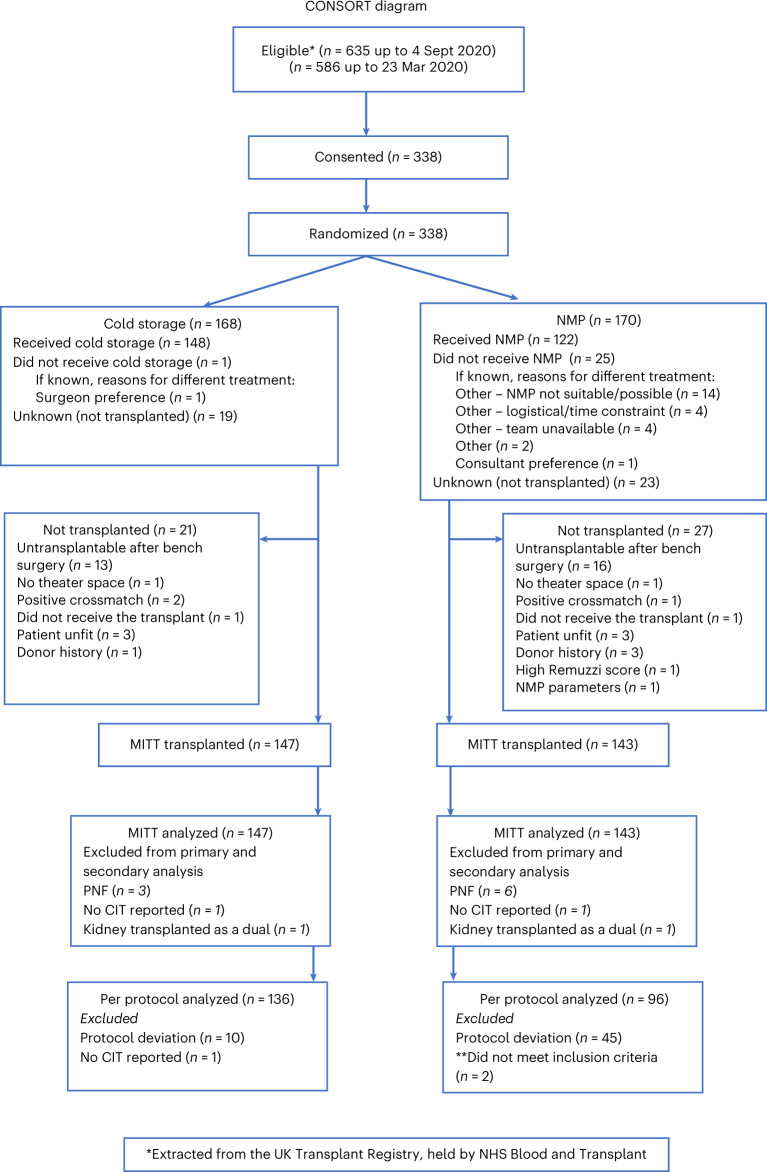
CONSORT diagram. Two eligibility periods are presented due to the COVID-19 pandemic: 4 September 2020 is the date the trial officially closed to recruitment, and 23 March 2020 was the start of the UK national lockdown. No participants were recruited between 23 March 2020 and 4 September 2020. Two participants did not have a cold ischemic time (CIT) reported, and two participants received dual transplants and, hence, do not have a left/right kidney variable populated; these four cases were excluded from all risk-adjusted modeling. **Two additional exclusion criteria (donors who underwent normothermic regional perfusion or one of a pair already randomized as a single kidney in the trial) were introduced on 13 October 2017, and, retrospectively, two participants fulfilled these exclusion criteria (both were randomized to NMP). These patients were included in the MITT analysis and excluded from the per-protocol analysis.

The characteristics of the donors and recipients are detailed in Table 1. All kidneys were from Maastricht category III or IV donors. From 13 February 2016 to 4 September 2020, there were 635 eligible DCD donor kidneys across the four UK centers (Fig. 1). In total, 338 patients were consented and randomized into the trial. Recruitment in all centers ceased on 23 March 2020 due to the Coronavirus Disease 2019 (COVID-19) pandemic. After reviewing the data, the Data Mangement Committee (DMC) made the recommendation that completing recruitment would not alter the primary outcome of the trial. In conjunction with the Trial Steering Committee (TSC), it was decided to officially end the trial on 4 September 2020. No additional patients were recruited between 23 March 2020 and 4 September 2020.

**Table 1:** Donor and recipient characteristics for the MITT analysis population

	SCS (*n* = 147)	NMP (*n* = 143)
Donor characteristics		
Age (years)	55 (46–64)	55 (48–65)
Male	88 (60%)	87 (61%)
Ethnicity		
White	139 (96%)	138 (97%)
Black	0 (0%)	0 (0%)
Asian	2 (1%)	3 (2%)
Other	4 (3%)	2 (1%)
BMI (kg/m^2^)	27 (24–31)	28 (24–33)
History of diabetes	13 (9%)	21 (15%)
History of hypertension	45 (31%)	51 (37%)
Recipient characteristics		
Age (years)	57 (48–66)	60 (51–66)
Male	90 (61%)	91 (64%)
Ethnicity		
White	120 (82%)	116 (81%)
Black	17 (12%)	16 (11%)
Asian	9 (6%)	8 (6%)
Other	1 (1%)	3 (2%)
History of diabetes	22 (15%)	15 (10%)
Dialysis status		
Pre-dialysis	32 (22%)	16 (11%)
Hemodialysis	87 (59%)	107 (75%)
Peritoneal dialysis	28 (19%)	20 (14%)
Previous transplant	20 (14%)	19 (13%)
Pre-transplant serum creatinine (µmol L^−1^)	567 (451–742)	584 (44–755)
Pre-transplant eGFR (ml/min/1.73 m^*2*^)	8 (6–11)	8 (6–11)
No HLA mismatches (% with no mismatches at the HLA-A, B or DR loci)	6 (4%)	1 (1%)
Kidneys with >1 artery	42 (28%)	40 (28%)
Left kidney transplant	69 (47%)	67 (47%)
Warm ischemic time (min)	18 (14–22)	17 (13–21)
Anastomosis time (min)	40 (34–50)	41 (33–50)
Total cold ischemic time (min)	828 (643–1027)	800 (594–986)
Mean first cold ischemic time (min)		649 ± 234
Mean second cold ischemic time (min)		130 ± 139

BMI, body mass index; HLA, human leucocyte antigen. The warm ischemic time is defined from the cession of circulation after withdrawal of life-sustaining treatment until the start of the in situ cold flush. The first cold ischemic time is calculated from the time of the in situ cold flush until the start of NMP. The second cold ischemic time is from the end of NMP when the kidney is flushed with cold preservation solution and placed back on ice until removal from ice for transplantation.

Summary of missing data: There was a small amount of missing data (fewer than 10 observations) for five characteristics: donor ethnicity, past diabetes, past hypertension, left kidney transplant and cold ischemic time. To note, 63 values were missing for pre- transplant eGFR, and 25 values were missing for duration of NMP.

Duplication of donors or recipients: 45 donors appear in the table twice (for left and right kidney).

Zero recipients appear in the table twice.

Data are *n* (%) for categorical variables and median (IQR) or mean ± s.d. for continuous variables. (The table excludes the 48 participants who were not transplanted.)

In total, 168 kidneys were allocated to SCS and 170 to NMP. In the SCS arm, one kidney received NMP due to the surgeon’s preference. In the NMP arm, 25 kidneys did not receive NMP. In 14 cases, this was due to the inability to secure a cannula into or around the renal artery; in eight cases, this was due to logistics with access to theater or time constraints; in one case, this was due to the surgeon’s preference; in one case, this was due to damage to the renal artery; and in one case, the kidney was transplanted as a dual transplant. A total of 21 kidneys in the SCS arm and 27 kidneys in the NMP arm were not transplanted (details are listed in Fig. 1). The final analysis included a total of 147 kidneys in the SCS arm and 143 in the NMP arm. No statistically significant difference was observed in the number of patients not transplanted in the two trial arms (*P* = 0.44; Fisher’s exact test).

There were no withdrawals from the trial. Two participants were randomized in error, as the single kidneys were transplanted as dual kidney transplants. Fifty-five participants experienced a protocol deviation: not receiving the randomized treatment was the most common reason (previously described); 21 kidneys that were one of a pair in the trial that were transplanted in the incorrect order to which they were randomized (due to logistics); and 13 kidneys that were randomized to NMP did not receive 60 min of NMP. All of the participants who experienced a protocol deviation were included in the modified intention-to-treat (MITT) analysis but were excluded from the per-protocol analysis. Four cases had to be excluded from all risk-adjusted modeling, as one of the risk adjustment factors was missing (two participants did not have a cold ischemic time reported, and two participants who received dual transplants did not have a left/right kidney variable populated) (Fig. 1). Details for missing data and protocol deviations are included in Supplementary Tables 1 and 2.

Two interim analyses were carried out, and the DMC recommended continuation after both (Supplementary Information).

### Primary outcome measure

In the MITT analysis, excluding PNF (SCS *n* = 3 and NMP *n* = 6), DGF occurred in 83 of 142 (58.5%) patients in the SCS arm and 82 of 135 (60.7%) patients in the NMP arm. No difference was observed between the arms (adjusted odds ratio (OR) (95% confidence interval (CI)) 1.13 (0.69–1.84), *P* = 0.62; Table 2). Similar rates were found in the per-protocol analysis (Table 2).

**Table 2:** Primary and secondary outcome measures

	Modified intention to treat		Per protocol	
Primary outcome	SCS (*n* = 147)	NMP (*n* = 143)	SCS (*n* = 136)	NMP (*n* = 96)
DGF *n* (%)	83/142 (58%)	82/135 (61%)	77/132 (58%)	54/92 (59%)
OR (95% CI) *P* value	1.13 (0.69–1.84) 0.624		1.08 (0.62–1.88) 0.798	
Secondary outcomes				
PNF *n* (%)	3/147 (2%)	6/143 (4%)	3/136 (2%)	3/96 (3%)
OR (95% CI)	2.34 (0.56–9.86)		1.95 (0.36–10.56)	
*P* value	0.233		0.441	
Duration of DGF (days)				
*n*	84	82	77	54
Median (IQR)	6 (2–9)	4 (1–9)	5 (1–9)	3 (1–8)
HR (95% CI)	0.97 (0.70–1.34)		0.98 (0.68–1.42)	
*P* value	0.866		0.917	
Functional DGF				
*n* (%)	45/53 (85%)	33/43 (77%)	42/50 (84%)	24/32 (75%)
OR (95% CI)	0.95 (0.29–3.10)		0.94 (0.27–3.26)	
*P* value	0.927		0.918	
Creatinine reduction ratio day 2% *n*	59	54	55	39
Mean ± s,d,	1.58 ± 16.5	7.97 ± 16.5	1.0 ± 16.3	8.9 ± 13.8
Mean difference (95% CI)	6.59 (0.49–12.7)		8.15 (1.6–14.7)	
*P* value	0.035		0.015	
Creatinine reduction ratio day 5% *n*	56	51	52	38
Mean ± s.d.	12.16 ± 26.61	23.76 ± 30.35	12.05 ± 25.99	23.13 ± 28.23
Mean difference (95% CI)	9.97 (−0.67–20.61)		8.88 (−2.75–20.51)	
*P* value	0.066		0.133	
Length of hospital stay (days) *n*	146	143	135	96
Median (IQR)	10 (7–15)	10 (7–16)	10 (7–15)	10 (7–18)
HR (95% CI)	0.91 (0.71–1.15)		0.90 (0.69–1.18)	
*P* value	0.428		0.433	
Biopsy-proven acute rejection				
Total number of events	23	36	23	25
Mean ± s.d.	0.2 ± 0.4	0.3 ± 0.7	0.2 ± 0.4	0.3 ± 0.7
Rate ratio (95% CI)	1.57 (0.83–2.95)		1.40 (0.71–2.76)	
*P* value	0.163		0.329	

Functional DGF was defined as a less than 10% reduction in serum creatinine levels for three consecutive days in the first week after transplantation. Creatinine reduction ratio day 2 and day 5 analyses excluded patients with PNF and DGF. Nine patients were excluded from the MITT cohort and six from the per-protocol cohort due to PNF. Mean ± s.d. or median (IQR) are unadjusted Kaplan–Meier estimates. HRs were calculated using Cox proportional hazards regression model. OR (95% CI) for PNF was calculated using a logistic regression model. ORs were calculated using a logistic regression model adjusted for cold ischemic time, donor age, left/right kidney and transplant center. Length of hospital stay was analyzed using Cox proportional hazards regression model. A negative binomial model was used to calculate the rate ratio (95% CI) of biopsy-proven acute rejection rates. *P* values were determined from the likelihood ratio test when including and excluding the treatment term from the model. Otherwise, *P* values were determined from the likelihood ratio test (two-sided test).

### Secondary outcome measures

Nine participants had PNF (three in the SCS arm and six in the NMP arm) and were excluded from subsequent analyses. In the SCS arm, two patients had a vascular thrombosis, and one had cortical necrosis. In the NMP arm, one patient had a vascular thrombosis; two patients had acute rejection; and, in three cases, the reason was unknown. No significant difference was observed in the number of patients who experienced PNF between the treatment arms for both the MITT analysis (adjusted OR (95% CI) 2.34 (0.56–9.86); Table 2) and the per-protocol analysis (adjusted OR (95% CI) 1.95 (0.36–10.56); Table 2).

Patients who experienced DGF were excluded from the outcomes and analyses for functional DGF, creatinine reduction ratio on post-transplant day 2 (CRR2) and creatinine reduction ratio on post-transplant day 5 (CRR5). The median duration of DGF was 6 (2–9) days in the SCS arm and 4 (1–9) days in the NMP arm. No significant difference was observed between the groups in the MITT (adjusted hazard ratio (HR) (95% CI) 0.97 (0.70–1.34); Table 2) or the per-protocol analyses (adjusted HR (95% CI) 0.98 (0.68–1.42); Table 2). The CRR on day 2 was significantly higher in the NMP arm in the MITT and per-protocol analysis (SCS 1.58 ± 16.5% versus NMP 7.97 ± 16.5%; adjusted mean difference (95% CI) 6.59% (0.5–12.7%), SCS 1.0 ± 16.3% versus NMP 8.9 ± 13.8%; adjusted mean difference (95% CI), 8.15 (1.6–14.7%), respectively; Table 2). By day 5, no significant difference was observed in the CRR nor in levels of functional DGF (Table 2). The length of hospital stay was also similar between groups in the MITT and per-protocol analyses (adjusted HR (95% CI) 0.91 (0.71–1.15) and 0.90 (0.69–1.18), respectively; Table 2).

The total number of patients who had biopsy-proven acute rejection was 19 in the SCS group and 24 in the NMP group. The unadjusted mean number of biopsy-proven acute rejection episodes per patient was numerically higher in the NMP arm compared to the SCS arm (0.3 per participant versus 0.2, respectively) but was not statistically different (adjusted rate ratio (95% CI) 1.57 (0.83–2.95) for the MITT population; Table 2 and Supplementary Table 3).

**Fig. 2:**
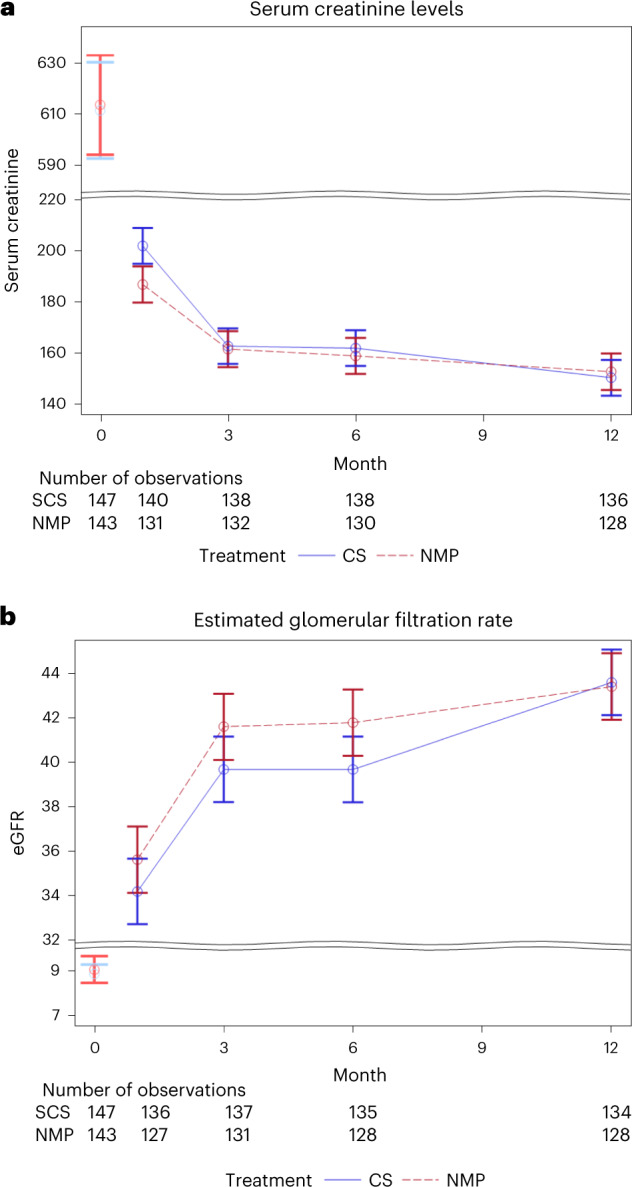
Renal function in renal transplant recipients receiving donor kidneys randomized to preservation by SCS or NMP. Serum creatinine levels (**a**) and eGFR levels (**b**) presented as unadjusted mean ± s.e.m. at baseline (timepoint 0) and adjusted mean ± s.e.m. for 1, 3, 6 and 12 months after transplant.

Serum creatinine and estimated glomerular filtration rate (eGFR) were not statistically significant at 1, 3, 6 or 12 months after transplant in the MITT or per-protocol analyses (*P* values from the time by treatment interaction term: serum creatinine 0.19 and 0.096, respectively, and eGFR *P* = 0.42 and *P* = 0.15, respectively; Supplementary Table 4 and Fig. 2a,b).

No differences were observed in tacrolimus trough blood levels between the SCS and NMP groups at 1, 3, 6 and 12 months after transplant (Supplementary Table 5).

### Patient and graft survival

**Fig. 3:**
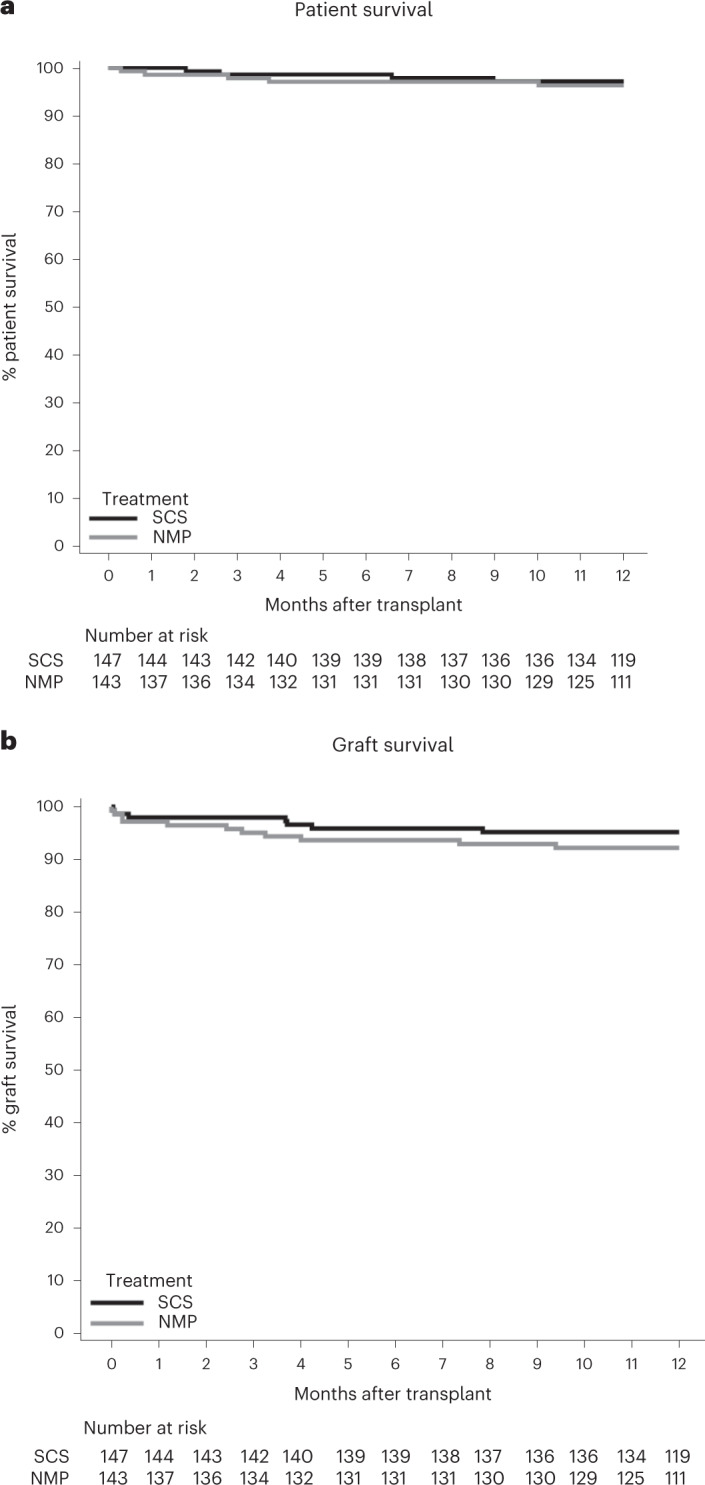
Patient and allograft survival in renal transplant recipients receiving donor kidneys randomized to preservation by SCS or NMP. Kaplan–Meier plot for 12-month patient survival (**a**) and graft survival (**b**).

Patient and graft survival were similar between the arms for both the MITT and per-protocol analyses (patient survival: SCS 97.2 versus NMP 96.3 (HR (95% CI) 1.44 (0.33–6.36)); Fig. 3a and Supplementary Table 4); (graft survival: SCS 95.2 versus NMP 92.2 (HR (95% CI) 1.47 (0.56–3.86)); Fig. 3b and Supplementary Table 4).

### Safety outcomes

For safety outcomes, the total number of incidences of biopsy-proven acute rejection, renal artery or venous thrombosis, complications of the renal transplant biopsy and the number of hospital admissions for any recognized complication of the renal transplant, including renal graft dysfunction, infection, surgery related or due to immunosuppression, were the same in both arms of the study (unadjusted mean SCS 0.7 ± 1.2 versus NMP 0.7 ± 1.2 (rate ratio (95% CI) 1.06 (0.73–1.52)) for the MITT analysis population; Table 3).

**Table 3:** Safety data analyses

Safety outcomes	Modified intention to treat		
Complication	SCS (*n* = 147)	NMP (*n* = 143)	Total (*n* = 290)
Renal artery thrombosis			
Total number of events	0	1	1
Mean (s.d.) per participant^a^	0 (0)	0 (0.1)	0 (0.1)
Rate ratio (95% CI)	Could not be estimated		
*P* value	Could not be estimated		
Renal vein thrombosis			
Total number of events	1	1	2
Mean (s.d.) per participant^a^	0 (0.1)	0 (0.1)	0 (0.1)
Rate ratio (95% CI)	Could not be estimated		
*P* value	Could not be estimated		
Complications of renal transplant biopsy			
Total number of events	3	3	6
Mean (s.d.) per participant^a^	0 (0.1)	0 (0.1)	0 (0.1)
Rate ratio (95% CI)	Could not be estimated		
*P* value	Could not be estimated		
Hospital admission for any recognized complication of renal transplantation and immunosuppression			
Total number of events	102	103	205
Mean (s.d.) per participant^a^	0.7 (1.2)	0.7 (1.2)	0.7 (1.2)
Rate ratio (95% CI)	1.06 (0.73–1.52)		
*P* value	0.7670		

^a^Unadjusted.

All rate ratios are from a negative binomial model adjusted for cold ischemic time, donor age, left/right kidney and transplant center. All *P* values (two-sided) are adjusted and are from the likelihood ratio test with and without the treatment effect.

For renal artery thrombosis, renal vein thrombosis and complications of renal transplant biopsy, very few events were observed in the trial, and, hence, it was not possible to undertake the modeling to assess for differences between the treatment arms. The number of hospital admissions for any recognized complication of renal transplantation (graft dysfunction, infection, surgery related) and immunosuppression was recorded. The number of events was compared using an adjusted negative binomial model.

### Exploratory assessment of kidney quality during NMP

The median renal blood flow was 180 ml/min/100 g (interquartile range (IQR) 120–230), and the median arterial pressure was 76 mmHg (IQR 74–80). The median amount of urine produced was 95 ml (IQR 50–180). The quality assessment score was applied to each of the analyzed kidneys. Forty-six percent had an assessment score of 2 of more. When adjusting for cold ischemic time, donor age, left/right kidney and transplant center, no significant difference in DGF was observed between those kidneys that scored a 1 versus 2 or more in the assessment score (adjusted OR (95% CI) 1.02 (0.47–2.24); Supplementary Table 6).

### Post hoc subgroup analysis of DGF

In a sub-analysis including nine PNF cases in the MITT cohort and six in the per-protocol cohort, no significant difference was observed in rates of DGF between the study arms (*P* = 0.510 and *P* = 0.722, respectively; Table 2 and Supplementary Table 7). In both subgroups, patients on dialysis or pre-dialysis, a smaller number of DGF events occurred within the NMP arm; however, this did not reach statistical significance, and no interaction was found between each group and dialysis (*P* = 0.90 for the interaction term; Supplementary Table 8). In the MITT analysis, if patients receiving a single dialysis session in the first 7 d after transplant were excluded, then DGF occurred in 70 of 147 (47.6%) patients in the SCS arm and 66 of 143 (46.2%) patients in the NMP arm. No difference was observed between the arms (*P* = 0.8149; Supplementary Table 9).

### Post hoc analysis of CRR2

In the MITT and per-protocol analysis, excluding patients who were not on dialysis before transplant, the CRR2 was significantly higher in the NMP arm (*P* = 0.0303 and *P* = 0.0067, respectively; Supplementary Table 10).

### Post hoc analysis of missing eGFR values

A sub-analysis imputing all missing eGFR values with the value 8.5 ml/min/1.73 m^2^ did not result in any significant differences between the groups in the MITT analysis (Supplementary Table 11).

### Post hoc analysis of the effect of a second period of cold ischemia on DGF rates after NMP

In the MITT analysis, no statistically significant difference was observed in the duration of the second cold ischemic time in NMP kidneys with initial graft function or DGF (median (range) 113.5 (1–514) minutes in NMP kidneys with initial graft function versus 134.6 (8–696) minutes in NMP kidneys with DGF; *P* = 0.4451; Supplementary Table 12). Second cold ischemic time had no effect on the rate of DGF (Supplementary Table 12).

## Discussion

We compared a 1-h period of NMP with conventional SCS for kidney transplantation from DCD donors. The NMP protocol had no effect on the primary endpoint, which was the incidence of DGF, defined as the requirement for dialysis in the first 7 d after transplant. No significant statistical differences were observed in the rates of acute rejection, renal function at 12 months, patient survival or graft survival. Our study also demonstrated that NMP is a safe procedure, as no significant differences were observed in complication rates when compared to SCS, and no adverse events were directly atrributable to NMP.

After the introduction of ex vivo NMP for donor kidneys into clinical practice^10^, the first non-randomized pilot study suggested that a 1-h period of NMP could substantially reduce the rate of DGF^17^. Other non-randomized studies of NMP have recently demonstrated DGF rates in the region of 30%, which was lower than expected for kidneys from this donor source^18–20^. In our trial, the rates of DGF were high, at around 60%, in both the NMP and SCS groups. These rates are not unusual and are similar to data previously reported in UK and European randomized trials of hypothermic machine perfusion (HMP) in DCD kidney transplantation^21–23^. Also, two recent large studies of controlled DCD kidney transplants in the Netherlands (*n* = 406 patients)^24^ and the UK (*n* = 225 patients)^25^ reported DGF rates of 67% and 65%, respectively, providing up-to-date contemporary data that are consistent with our trial outcomes. Although lower rates of DGF in DCD kidney transplants have been reported in the UK, these were derived from registry data, which are incomplete and of variable quality compared to the actuality of data collected in the setting of a prospective clinical trial^2^.

There are several possible causes of the high rates of DGF described here and in some of the previous literature. Physiological parameters during the agonal period, defined as the time between withdrawal of treatment and circulatory arrest, are likely to be critical. In the UK, withdrawal of treatment comprises disconnection from the ventilator and stopping inotropes but, in many centers, does not include removal of the endotracheal tube. Continuing support of the airway in this way can prolong the agonal period. Even more importantly, severe and prolonged hypotension during the agonal period and the consequent inadequate organ perfusion causes additional warm ischemic injury and has been shown to be associated with higher DGF rates in DCD kidneys^24^. We were not able to study the effects of hypotension after the withdrawal of life-sustaining treatment in our trial because the blood pressure during the agonal phase is not routinely recorded in the UK.

The high rates of DGF reported here are also related to defining DGF as the requirement for dialysis in the first 7 d after transplant. Although this is the simplest and most widely used definition of DGF^26^, it does not consider severity. In particular, this definition includes patients who undergo a single early postoperative dialysis for hyperkalemia or fluid overload, irrespective of their initial graft function. This overestimates the rate of clinically relevant DGF because there is evidence that a single postoperative dialysis has no effect on long-term transplant outcomes^27^. If patients receiving a single postoperative dialysis were excluded from the analysis, the rates of DGF in both treatment arms were less than 50%, but there were still no significant differences between the SCS and NMP groups.

The use of normothermic regional perfusion (NRP), which restores oxygenated blood flow to the abdominal organs in situ after cardiac arrest, has yielded even lower DGF rates of 23–30%^28,29^. Although these results are notable, the effects of NRP have yet to be investigated in a randomized controlled trial, and the series reported may have an element of selection and observation bias. In our trial, NRP was a specific exclusion criterion to remove it as a potentially confounding variable.

Conventional SCS was used as the control arm in this study because it is the standard of care in the UK. Although HMP has been shown to reduce the rate of DGF in DCD kidneys when compared to SCS^21,30,31^, it has not been widely adopted in many countries, including the UK. Comparison of NMP and HMP is an important future direction, and there is currently a trial ongoing to address this (ClinicalTrials.gov identifier: NCT04882254)^32^.

There are several possible explanations for the lack of effect of NMP on DGF rates. A 1-h period of NMP may not be long enough to reverse the effects of cold ischemia on renal tubular cells. The rationale for our trial design was based on pilot clinical data suggesting that 1 h of NMP could significantly reduce the rate of DGF in DCD kidneys^17^. Further justification for a short period of NMP was based on experimental work using porcine kidneys, which demonstrated that brief periods of NMP restored depleted cellular ATP levels^12^. This was reinforced by a study of human kidneys declined for transplantation that showed an increase in the expression of glycolytic pathway and oxidative phosphorylation genes, suggesting that NMP has the potential to increase cellular capacity to generate ATP and restore homeostasis^33^.

There is limited evidence to define the optimal duration of NMP. One experimental study suggested that 1 h of NMP is not as beneficial as more prolonged periods of perfusion^34^. The feasibility of a 24-h period of NMP has been demonstrated using a urine recirculation protocol in discarded human kidneys^35^, but there are currently no published clinical studies of prolonged NMP.

The original intention in our protocol was to deliver a short period of NMP immediately before the kidney was transplanted, but this was not always achieved because of the logistics of preparing patients for transplant surgery. After 1 h of NMP, the kidney was re-flushed with cold preservation solution and placed back on ice for a variable second period of cold storage until removal from ice for implantation. The mean duration of this second cold time was just over 2 h, and, although this may not influence outcome, longer additional cold storage would eventually counteract any beneficial effects of NMP. Post hoc analysis demonstrated that there was no difference in the duration of the second cold ischemic period in NMP kidneys with or without DGF.

In liver transplantation, donor livers have been maintained by NMP for the full duration of the preservation period^13^. However, this presents an appreciable logistical challenge, and, currently, the most common practice is to transport livers in cold storage and then perform NMP for a few hours before transplantation^36^. Future studies in kidney transplantation will need to address the place of more prolonged periods of NMP using protocols similar to the current practice in liver transplantation.

The induction of inflammation during NMP is another potential reason for the lack of its effect on the rate of DGF. The red-cell-based perfusate used in the NMP system was designed to create an anti-inflammatory environment without platelets, white cells or complement^37^. Nonetheless, global transcriptomic studies have clearly demonstrated the opposite effect, with upregulation of several immune and inflammatory pathway genes in human kidneys. More detailed analysis of the transcriptomics data demonstrated that kidneys with a lower expression of oxidative phosphorylation pathways and enhanced upregulation of inflammatory cytokines and chemokines were more likely to have longer periods of DGF compared to those that had immediate graft function or just 1 d of DGF^33^. We have also shown that the addition of a cytokine filter to the NMP circuit attenuates the expression of inflammatory genes in human kidneys, and this has potential for clinical application in the future^33^.

Our trial has several limitations. It was designed as an open-label study because of the logistics of the NMP technique. Blinding of the surgical team was not possible because NMP was performed in the operating room while the transplant recipient was being prepared for surgery. However, the perfusion teams were not involved in data analysis. We were unable to perform NMP in 14 kidneys (8.2%) randomized to this treatment arm because of concerns over the technical aspects of cannulating the renal arterial system. The alternatives for arterial perfusion are the use of an aortic patch clamp or direct cannulation of the renal artery. The former is more favorable as it allows perfusion of multiple renal arteries without loss of the aortic patch for subsequent anastomosis in the recipient^38^. Nonetheless, complex renal arterial anatomy or heavily diseased aortic patches sometimes makes cannulation impossible or increases the risk of causing damage such as an arterial dissection. In this early experience, we took a cautious approach and chose not to perform NMP in higher-risk circumstances. More complex arterial perfusion techniques, such as anastomosing the renal arterial system to a tissue-banked deceased donor artery to create a discardable conduit for perfusion, are possibilities in the future that could increase the rate of implementing NMP. The intention-to-treat analysis takes account of the non-performance of NMP on the outcome of the trial.

As NMP requires cannulation of the renal blood vessels, there is potential to cause endothelial damage. There is also a risk of transmitting infection during the period in which the kidney is perfused in an ex vivo organ chamber. In our trial, NMP was not associated with any increase in transplant thrombosis, infectious complications or any other adverse events. No significant differences were observed between the groups in terms of recipient safety outcomes. The incidence of renal arterial or venous thrombosis was very low, and there were few complications related to the renal transplant biopsies. Complications associated with kidney transplantation that require hospitalization are common and were categorized into graft dysfunction, infection, related to surgery or immunosuppression based.

In conclusion, a 1-h period of NMP after SCS does not reduce the risk of DGF in DCD kidney transplants. Nonetheless, we have demonstrated that this new technology for kidney preservation is feasible, safe and suitable for clinical application. This trial delivers the first, essential step in exploring the broader potential of NMP in kidney transplantation.

## Methods

### Trial design

This investigator-led, randomized controlled, open-label trial was approved by the UK National Research Ethics Service and local institutional review boards (REC 15/EE/0356), with trial registration number ISRCTN15821205. An independent DMC monitored the progress of the trial. The trial design and methods were published previously^39^.

### Trial patients

Eligible patients enrolled on the transplant waiting list and allocated a suitably matched kidney were enrolled at four UK transplant centers. Inclusion criteria included recipients 18 years of age or older with end-stage renal failure requiring their first or second kidney transplant who received a kidney from Maastricht category III or IV DCD donors 18 years of age or older. Exclusion criteria included recipients receiving a third or subsequent kidney transplant, multi-organ transplants, dual kidney transplants, pediatric en bloc kidney transplants and kidneys preserved by HMP. Two additional exclusion criteria were introduced on 13 October 2017: donors who underwent normothermic regional perfusion or one of a pair already randomized as a single kidney in the trial. This was approved by the Research Ethics Service, local institutional review boards and the TSC. All patients provided written informed consent.

Patients were randomly assigned in a 1:1 ratio to 1-h NMP or SCS. The randomization list was created by the trial statistician in SAS Enterprise Guide (version 5.1) with SAS 9.4, stratified by transplant center and using randomly permuted blocks of fixed size 2 and 4 for single and pairs of kidneys, respectively. In cases where paired kidneys from the same donor were transplanted in the same transplant center, the randomization was stratified by kidney (right or left) so that one kidney was randomly allocated to each treatment and in which order they should be transplanted. The randomization process was facilitated using an Interactive Web Response System. After the assignment of treatment arms, no one in the trial was blinded to the treatment allocation.

### SCS

Kidneys were retrieved by National Health Service Blood and Transplant (NHSBT) National Organ Retrieval Service teams, and, after flushing with cold preservation solution, they were stored on ice until transplanted.

### NMP

NMP was performed at the transplanting center for 1 h using a customized pediatric cardiopulmonary bypass system. Kidneys were perfused with an oxygenated red-cell-based solution supplemented with a crystalloid solution and amino acids. Details are documented in the Supplementary Information. After the 1-h period of NMP, kidneys were flushed with hyperosmolar citrate solution at 4 °C to remove the red-cell-based perfusate and to re-cool the organ before transplantation. NMP kidneys were stored in ice and transplanted as soon as possible.

### Transplantation

Kidneys were transplanted into either iliac fossa with anastomosis of the artery to the common, external or internal iliac arteries. The vein was anastomosed to either the common or the external iliac vein. The ureteric anastomosis was performed as an extravesical onlay over a double J stent.

### Immunosuppression

A standard immunosuppressive protocol was used in all four trial centers. All patients received induction therapy with basiliximab 20 mg IV given on the day of transplantation and on the fourth postoperative day. All patients received methylprednisolone 500 mg IV at induction of anaesthesia. Maintenance immunosuppression was given as triple therapy with tacrolimus, mycophenolic acid and prednisolone. Tacrolimus was administered at a dose of 0.1 mg/kg/day orally either in two divided doses (adoport) or as a single daily dose (advagraf). Tacrolimus trough blood levels were measured at least twice weekly, and the therapeutic target range in the first 3 months after transplant was 5–10 ng ml^−1^. Levels were analysed at 1, 3, 6 and 12 months after transplant. Patients received mycophenolate mofetil (CellCept) 500 mg twice daily orally and prednisolone 20 mg once daily orally. The dose of prednisolone was tapered to 5 mg once daily by 2–6 weeks after transplantation.

### Clinical outcomes

The primary outcome measure was DGF, defined as the requirement for dialysis in the first week after transplantation. Secondary outcome measures included incidence of PNF; duration of DGF; functional DGF defined as less than 10% fall in serum creatinine for three consecutive days in the first week after transplantation; CRR2 (creatinine day 1 − creatinine day 2 / creatinine day 1 × 100), CRR5 (creatinine day 1 − creatinine day 5 / creatinine day 1 × 100); duration of hospital stay; serum creatinine and eGFR at 1, 3, 6 and 12 months using the Modification of Diet in Renal Disease (MDRD) 4 variable equation; and patient and allograft survival up to 12 months after transplant. For safety outcomes, the total number of incidences of biopsy-proven acute rejection, renal artery or venous thrombosis, complications of the renal transplant biopsy and the number of hospital admissions for any recognized complication of the renal transplant, including renal graft dysfunction, infection, surgery related or due to the immunosuppression, were recorded.

Data were collected by each of the participating transplant centers using an online secure database hosted by the NHSBT Clinical Trials Unit.

### Statistical analysis

#### Study design

The NHSBT Clinical Trials Unit supported the design, data management and analysis of the trial. Historical data spanning a 5-year period for three participating centers showed that the overall rate of DGF in DCD kidney transplants was 50%. This was used as the baseline rate. In a pilot series of kidney transplants from extended criteria donors (ECDs), 18 kidneys undergoing SCS followed by 1 h of NMP were compared to a historical control group of 47 ECD transplants after SCS alone. The DGF rates were 1/18 (6%) in the NMP group compared to 17/47 (36%) in the SCS group. Using a fixed sample size study, with interim analyses after 124 and 248 participants had been enrolled and reached 7 d after transplant, a total of 370 patients receiving a DCD kidney were required to detect a 30% relative reduction in DGF (from 50% to 35%) with a power of 80%, a statistical significance of α = 0.05 and 1:1 allocation. To allow for a study withdrawal rate of 7.5%, a maximum of 400 patients were needed for recruitment. There would be no sample size re-estimation during the trial.

### Interim analysis

A group sequential design, with O’Brien–Fleming stopping rules (which preserved the 5% significance level in the final analysis), was used to allow the DMC to review the primary outcome for evidence of harm, benefit or futility. Two unadjusted interim analyses were performed—the first after 124 patients were randomized and reached 7 d after randomization and the second after 248 patients were randomized and reached 7 d after randomization. The stopping rules were used as a guideline, alongside the other safety data available to the DMC, as an overall assessment of the trial. The interim analyses were performed by the trial statistician who was unblinded to the treatment arm, and these results were presented to the DMC only. The DMC reported its recommendations, without disclosing any trial results, to the TSC, which made the final decision regarding continuation of the trial.

### Study population

The population used for efficacy analyses was a MITT population including all randomized patients who received a transplant. This was a change from the original protocol because it was deemed illogical to include those participants who did not receive a transplant, as no outcome data were available. Primary and secondary outcomes were also analyzed per protocol, which excluded any participant who did not receive a transplant, was randomized in error, experienced a protocol deviation or was withdrawn from the trial (details are provided in the statistical analysis plan). For both analysis populations, results were presented by randomized treatment, and all ratios and mean differences were presented as NMP versus SCS.

All analyses were adjusted for cold ischemic time, donor age, left/right kidney and transplant center (all as fixed effects). All tests were two-sided, and *P* values less than 0.05 were considered statistically significant. SAS Enterprise Guide (version 7.15) with SAS 9.4 was used to conduct all analyses. It was pre-specified in the statistical analysis plan that multiple comparisons would be performed, potentially increasing the probability of observing a statistically significant result by chance, but that no adjustments would be made to account for multiple testing.

### Primary and secondary outcome measures

The primary outcome was analyzed using an adjusted logistic regression model and excluded participants who experienced PNF. The data for this outcome were complete, and, therefore, it was not necessary to undertake any of the methods proposed in the statistical analysis plan for assessing the impact of these missing data.

Secondary and safety outcome measures were analyzed using logistic regression model (PNF and functional DGF), Cox proportional hazards model (duration of DGF, length of hospital stay, allograft and patient survival), normal linear regression model (CRR at day 2 and day 5, serum creatinine and eGFR at 1, 3, 6 and 12 months) and negative binomial model (biopsy-proven acute rejection and safety outcomes). Missing secondary outcome measures were not imputed and were excluded from the relevant analyses, except for eGFR. Full details can be found in the statistical analysis plan. To ensure model assumptions were met, residual plots were examined.

### NMP assessment score

After NMP, kidneys were allocated a score of 1–5 based on the macroscopic appearance, mean renal blood flow and urine production. A lower value indicated a better score (details in the Supplementary Information). To assess for associations between DGF and the NMP assessment score, a logistic regression model was fitted, adjusting for cold ischemic time, donor age, left/right kidney and transplant center. Participants who experienced PNF were excluded from the analysis. The NMP assessment score was fitted as a binary variable.

### Post hoc subgroup analyses

i. PNF was included in the DGF groups to determine the impact of the pre-transplant preservation interventions on rates of non-function.
ii. To determine the effect of pre-transplant recipient dialysis status (receiving dialysis versus pre-dialysis) on DGF in the MITT analysis, we used the same model as that used for the primary outcome but with the inclusion of the pre-transplant dialysis term, and we also assessed the interaction between treatment group and pre-dialysis status.
iii. Some patients received a single post-transplant dialysis as a safety measure in response to hyperkalemia or fluid overload, irrespective of renal function. To take account of this, we analyzed the effect of excluding patients who received a single post-transplant dialysis on DGF rates.
iv. We also analysed the effect of excluding pre-dialysis patients from CRR2 calculations in both the MITT and per-protocol analyses.
v. The duration of the second cold ischemic period after NMP was variable, and this might have influenced the rate of DGF. We, therefore, compared the duration of second cold ischemic time in kidneys with initial function and DGF after NMP.
vi. To take account of missing eGFR data in the MITT analysis, we imputed a value of 8.5 ml/min/1.73 m^2^ at the 1-, 3-, 6- and 12-month timepoints for patients with PNF, ongoing DGF, graft loss or death^40^.

### Reporting Summary

Further information on research design is available in the Nature Portfolio Reporting Summary linked to this article.

## Online content

Any methods, additional references, Nature Portfolio reporting summaries, source data, extended data, supplementary information, acknowledgements, peer review information; details of author contributions and competing interests; and statements of data and code availability are available at 10.1038/s41591-023-02376-7.

## Supplementary information

Supplementary Information Text, tables, protocol and statistical plan.

Reporting Summary

## Data Availability

Data from the trial are stored in an online secure database hosted by the NHSBT Clinical Trials Unit. The protocol, consent form, statistical analysis plan, definition and derivation of clinical characteristics and outcomes, training materials, regulatory documents and other relevant study materials are available online and were published elsewhere. The datasets generated during analysis will be available upon reasonable request from the NHSBT Clinical Trials Unit after de-identification (text, tables, figures and appendices) 9 months after publication and ending 5 years after article publication. Data will be shared with investigators whose use of the data has been assessed and approved by an NHSBT review committee as a methodologically sound proposal. The NHSBT Clinical Trials Unit can be contacted at CTU@nhsbt.nhs.uk. The Clinical Trials Unit will be able to provide a copy of our data-sharing policy and arrange a data use agreement, which will need to be signed. All data use agreements will be in line with the consent given by participants upon agreeing to take part in the trial.
