# Supplementary material for: Normothermic machine perfusion versus static cold storage in donation after circulatory death kidney transplantation: a randomized controlled trial
Source: Nat Med. 2023 May 25;29(6):1511–9. doi: 10.1038/s41591-023-02376-7 (PMC10287561; doi:10.1038/s41591-023-02376-7)
Supplement: Supplementary file 1 — Text, tables, protocol and statistical plan. [file 41591_2023_2376_MOESM1_ESM.pdf]

nature medicine

Article

https://doi.org/10.1038/s41591-023-02376-7

Normothermic machine perfusion versus static cold storage in donation after circulatory death kidney transplantation: a randomized controlled trial

In the format provided by the authors and unedited

SUPPLEMENTARY INFORMATION

Normothermic machine perfusion versus static cold storage in donation after circulatory death kidney transplantation: a randomised controlled open-label trial

List of investigators and study sites

Addenbrookes Hospital, Cambridge – Miss Irum Amin, Mr Mazin Hamed, Mr Simon Harper, Mr Vasilis Kosmoliaptsis, Professor Gavin Pettigrew, Mr Neil Russell, Professor Kourosh Saeb-Parsy, Mr Dominic Summers, Miss Ann-Marie O'Sullivan, Mrs Julia Ertner

Guy's Hospital, London – Mr Nicholas Barnett, Mr Francis Calder, Mr Pankaj Chandak, Mr Martin Drage, Mr Nikolaos Karydis, Mr Nicos Kessaris, Mr Ioannis Loukopoulos, Professor Nizam Mamode, Mr Jonathon Olsburgh, Miss Kiran Sran, Miss Rhana Zakri

Freeman Hospital, Newcastle upon Tyne – Mr Rodrigo Figueiredo, Mr Jeremy French, Mr John Hammond, Mr Ibrahim Ibrahim, Professor Derek Manas, Mr Sanjay Pandanaboyana, Mr David Rix, Mr Gourab Sen, Professor David Talbot, Miss Emily Thompson, Professor Steve White

Edinburgh Royal Infimary – Miss Sorina Cornateanu, Mr John Terrance, Mr Avinash Sewpaul, Mr Andrew Sutherland, Mrs Fiona Hunt

Perfusionists – Corrina Fear, Keziah Crick and Sharayne Robinson

NHSBT Clinical Trials Unit – Alison Deary, Dave Collett, Helen Thomas, Rebecca Hendry, Cara Hudson, Lekha Bakrania, Sioban Martin and Valerie Hopkins

Data Monitoring Committee (DMC) – Professor Peter Friend, Professor Paul White and Dr Chris Dudley

Trial Steering Committee (TSC) – Professor Lorna Marson and Professor Grant Stewart

Mr Rithin Punjala at Guy's Hospital and Lesley Coutts and Lynsey Farwell at the Edinburgh Royal Infirmary for helping with normothermic machine perfusion.

Supplementary methods

Normothermic machine perfusion

The normothermic machine perfusion (NMP) circuit was designed using paediatric cardiopulmonary bypass technology (Medtronic, Watford, UK) and consisted of a centrifugal blood pump (Bio-pump 560), a heat exchanger, a venous reservoir (Medtronic), 1/4 inch PVC tubing and an Affinity membrane oxygenator (Medtronic). The hardware included a speed controller, a TX50P flow transducer and a temperature probe (Cole-Parmer, London, UK). Two infusion pumps were also incorporated into the system.

Perfusate

The circuit was primed with perfusate solution (Ringer's solution, Baxter Healthcare, Thetford, Norfolk, UK, 400mL) and one unit of either O positive or O negative packed red cells from the local blood bank. Mannitol 10% 15mL (Baxter Healthcare, Thetford, UK), dexamethasone 8 mg (Organon Laboratories, Cambridge, UK) and heparin 3000iu (CP Pharmaceuticals, Wrexham, UK) was added to the perfusate. Sodium bicarbonate 8.4% (Fresenius Kabi, Cheshire, UK) was added to normalise the pH. A nutrient solution (Synthamin 17, Baxter Healthcare) with sodium bicarbonate 8.4% 15mL, insulin 100iu (Novo Nordisk, Denmark) and multivitamins 5mL (Cernevit, Baxter Healthcare) was infused into the circuit at a rate of 20 mL/h. Prostacyclin 0.5 mg (Flolan, Glaxo-Wellcome, Middlesex, UK) was infused into the arterial arm of the circuit at a rate of 4 mL/h and glucose 5% (Baxter Healthcare) at 4 mL/h. Ringer's solution was used to replace urine output mL for mL.

Perfusion

Kidneys were placed in a sterile perfusion chamber and the renal artery was cannulated with either a straight cannula or patch clamp to preserve the aortic patch. The arterial cannula was primed with cold 0.9% sodium chloride to exclude any air from the circuit at the start of perfusion. The renal vein was either cannulated for recirculation of the perfusate or the effluent from the vein left to drain back freely

from the chamber into the reservoir. The surgical teams involved in the study were given the choice of cannulating renal veins or leaving them free to take account of short renal veins or complex venous anatomy.

Kidneys were perfused at a set mean arterial pressure (70–85 mmHg). The plasma-free red cell-based perfusate was circulated from the venous reservoir through the centrifugal pump into the membrane oxygenator where it was oxygenated (95% oxygen/5% carbon dioxide) and warmed to 35–37°C. The perfusate then flowed through the arterial limb of the circuit to the renal artery. Venous return from the renal vein was fed back into the reservoir.

Renal blood flow (RBF) was monitored every 5 minutes during NMP. The total urine output was recorded. Blood gas analysis was used to measure the acid–base balance pre- and post-NMP.

After NMP, kidneys were flushed with approximately 500 to 1000 mL of cold (4°C) hyperosmolar citrate (HOC, Baxter Healthcare, UK) to remove the perfusate and then placed back in ice until transplanted.

NMP assessment score

An assessment score was recorded for all kidneys undergoing NMP.[1] A visual assessment of the macroscopic appearance of the kidney was made throughout the 1h of NMP. In addition, the mean RBF and total urine output was measured over 1h of NMP. Scores were attributed as follows:

Macroscopic perfusion:

Excellent perfusion with global and even pink appearance (1 point)

Moderate perfusion with some areas of patchy or mottled perfusion (2 points)

Poor perfusion with a globally mottled and purple appearance (3 points)

Renal blood flow (RBF):

RBF <50mL/h/100g (1 additional point)

Urine output (UO):

UO <43mL/h (1 additional point)

Scores for macroscopic appearance, RBF and UO were added to yield an overall assessment score ranging from 1 (the highest quality) to 5 (the lowest quality).

Supplementary results

Interim analysis results

The first interim analysis of 137 patients was carried out in March 2018. One hundred and ten kidneys had been transplanted (54 static cold storage (SCS) and 56 NMP) and reached the primary end point. The rate of DGF was 61% in the SCS arm and 71% in the NMP arm. The standardised Z score, from the group sequential analysis with O'Brien–Fleming stopping rules, was calculated as –1.11. This was outside the acceptance or rejection regions and the stopping rules suggested that the trial should continue. The Data Monotioring Committee (DMC) agreed with this decision. The second interim analysis of 251 patients was carried out in June 2019. Two hundred and sixteen kidneys had been transplanted and reached the primary end point. The rate of DGF was 58% in the SCS arm and 61% in the NMP arm. The standardised Z score was calculated as –0.53. This was inside the futility region and suggested that the trial should be stopped. The DMC requested that the analysis was replicated per protocol, excluding those participants who did not receive the intended treatment (n = 14). The revised rate of DGF was 58% in the CS arm and 60% in the NMP arm. The standardised Z score was calculated as –0.28. This was inside the futility region and the stopping rules suggested that the trial should be stopped. Nonetheless, the DMC concluded that the trial should continue because the result was borderline and there were no safety concerns with the trial.

References

1. Hosgood SA, Thompson E, Moore T, Wilson CH, Nicholson ML. Normothermic machine perfusion for the assessment and transplantation of declined human kidneys from donation after circulatory death donors. *Br J Surg* 2018; **105**: 388–94.

Table 1. Summary of the missing primary and secondary outcome data. All transplanted participants had the primary outcome measure populated and hence there did not need to be any imputation of this variable. If the data item was not expected (e.g. a participant died before the 3 month follow up, the serum creatinine at 3 months would not be expected) or was not required for an outcome (e.g. when looking at the primary outcome and a participant experienced primary non-function (PNF), the primary outcome was not required), these cases have been excluded from the numbers and percentages below. Data excludes participants who were not transplanted (SCS n = 21 and NMP n = 27).

**Missing data table n (% of total expected)**			
	**SCS (n=147)**	**NMP (n=143)**	**Total (n=290)**
Missing primary outcome	0 (0)	0 (0)	0 (0)
Imputed primary outcome	N/A	N/A	N/A
Missing PNF information	0 (0)	0 (0)	0 (0)
Missing duration of DGF	1 (1)	1 (1)	2 (1)
Missing functional DGF information	6 (10)	11 (20)	17 (15)
Missing CRR day 2 (CRR2)	0 (0)	0 (0)	0 (0)
Missing CRR day 5 (CRR5)	3 (5)	3 (6)	6 (5)
Missing length of hospital stay	1 (1)	0 (0)	1 (0)
Missing biopsy-proven acute rejection episodes*	0 (0)	0 (0)	0 (0)
Missing serum creatinine			
At 1 month	0 (0)	0 (0)	0 (0)
At 3 months	1 (2)	1 (2)	2 (2)
At 6 months	0 (0)	1 (2)	1 (1)
At 12 months	0 (0)	0 (0)	0 (0)
Missing eGFR			
At 1 month	2 (3)	1 (2)	3 (3)
At 3 months	1 (2)	1 (2)	2 (2)
At 6 months	1 (2)	1 (2)	2 (2)
At 12 months	1 (2)	0 (0)	1 (1)
Missing 12-month patient survival	0 (0)	0 (0)	0 (0)
Missing 12-month graft survival	0 (0)	0 (0)	0 (0)

*As the rejection data collection form was only completed if the participant experienced rejection, the assumption was made that if the form was not present then the participant did not experience any biopsy-proven rejection.

CRR: creatinine reduction ratio; DGF: delayed graft function; eGFR: estimated glomerular filtration rate; NMP: normothermic machine perfusion; SCS: static cold storage.

Table 2. Protocol deviations in the static cold storage (SCS) and normothermic machine perfusion (NMP) groups. A total of 55 participants (10 randomised to SCS and 45 randomised to NMP) experienced at least one protocol deviation. All participants with protocol deviations were included in the modified intention-to-treat analysis where the outcome was available. Some participants experienced more than one protocol deviation.

**Protocol deviations** (excluding all participants who were not transplanted)		
Detail of protocol deviation	SCS	NMP
Did not receive randomised treatment. Pair of kidneys transplanted in the incorrect order to which they were randomised	1	4
Did not receive 60 minutes of NMP		13
Did not receive randomised treatment		21
Pair of kidneys transplanted in the incorrect order to which they were randomised	9	7

Table 3. BANFF classification for biopsy-proven rejection episodes for the modified intention-to-treat analysis population and per protocol populations.

	**Modified intention-to-treat**		**Per protocol**	
	SCS (n=147)	NMP (n=143)	SCS (n=136)	NMP (n=96)
**Biopsy-proven rejection episodes**				
n	23	36	23	25
**BANFF classification**				
Borderline	1	0	1	0
Grade IA	6	9	6	4
Grade IB	1	4	1	3
Grade IIA	5	5	5	3
Grade IIA+AMR	1	1	1	0
Grade IIB	1	4	1	2
Grade IIB+AMR	1	0	1	1
Grade III	0	1	0	1
Unknown	7	12	7	11

NMP: normothermic machine perfusion; SCS: static cold storage. AMR: antibodymediated rejection.

Table 4. Results from the modified intention-to-treat and per protocol analysis populations of serum creatinine and estimated glomerular filtration rate (eGFR) at 1, 3, 6 and 12 months post-transplant, and patient and graft survival in the static cold storage (SCS) and normothermic machine perfusion (NMP) arms of the trial. N = patients with outcome completed. All mean differences, P values and hazard ratios have been adjusted for cold ischaemic time, donor age, left/right kidney indicator and centre. For serum creatinine and eGFR outcomes, values excluded patients who had primary non-function and if the participant's kidney failed prior to the follow-up timepoint they were not included in the analysis at the subsequent timepoint. Mean values of serum creatinine and eGFR are unadjusted and mean difference was calculated using adjusted normal linear regression model with random effect. The P values (two-sided) are from the likelihood ratio test when including and excluding the time by treatment interaction term from the model. Patient and graft survival are unadjusted Kaplan–Meier estimates and hazard ratios (HR) (95% confidence interval (CI)) were calculated using an adjusted Cox proportional hazards regression model. P value obtained from the log-rank test (two-sided test) when Kaplan-meier was used.

	**Modified intention-to-treat**		**Per protocol**	
	SCS (n=147)	NMP (n=143)	SCS (n=136)	NMP (n=96)
**Serum creatinine (µmol/L)**				
1 month (n)	140	131	129	89
Mean ± SD	201 ± 118	186 ± 79	199 ± 119	184 ± 69
3 months (n)	138	132	129	90
Mean ± SD	163 ± 66	163 ± 66	163 ± 67	164 ± 63
6 months (n)	138	130	129	90
Mean ± SD	162 ± 65	158 ± 64	163 ± 67	159 ± 59
12 months (n)	136	128	127	89
Mean ± SD	148 ± 56	151 ± 61	148 ± 57	156 ± 57
**Mean difference at**	–15.04 (–32.58–2.50)		–16.40 (–36.49–3.70)	
1 month	–1.15 (–18.69–16.40)		–0·67 (–20.73–19.39)	
3 months	–3.02 (–20.62–14.57)		–1.99 (–22.07–18.08)	
6 months	2.37 (–15.30–20.04)		8.28 (–11.87–28.43)	
12 months	0.19		0.10	
P value				

**eGFR (mL/min/1.73m^2)**				
1 month (n)	136	127	126	87
Mean ± SD	35 ± 16	37 ± 16	35 ± 16	36 ± 15
3 months (n)	137	131	128	90
Mean ± SD	40 ± 16	42 ± 17	40 ± 16	41 ± 16
6 months (n)	135	128	126	89
Mean ± SD	40 ± 17	43 ± 16	40 ± 17	42 ± 15
12 months (n)	134	128	125	89
Mean ± SD	45 ± 19	44 ± 18	45 ± 19	42 ± 16
**Mean difference at**<br>1 month<br>3 months<br>6 months<br>12 months<br>P value	 1.43 (–2.24–5.10)<br>1.92 (–1.74–5.57)<br>2.10 (–1.57–5.77)<br>–0.19 (–3.86–3.49)<br>0.42		 1.00 (–3.16–5.17)<br>1.24 (–2.90–5.38)<br>1.99 (–2.17–6.14)<br>–1.74 (–5.90–2.42)<br>0.15	
**Patient survival (n)**	147	143	136	96
Probability of patient survival after 12 months	97.2	96.3	98.5	98.9
HR (95% CI)	1.44 (0.33–6.36)		0.64 (0.04–9.65)	
**Graft survival (n)**	147	143	136	96
Probability of graft survival after 12 months	95.2	92.2	94.8	93.8
HR (95% CI)	1.47 (0.56–3.86)		1.11 (0.36–3.44)	

SD: standard deviation.

Table 5. Trough tacrolimus levels at 1, 3, 6 and 12 months post-transplant for transplanted patients only. Values are median (interquartile range (IQR)).

	**Modified intention-to-treat**		**Per protocol**	
	SCS (n=147)	NMP (n=143)	SCS (n=136)	NMP (n=96)
Trough level				
1 month (n)	139	136	128	93
Median (IQR)	8.8 (6.8–11)	8.2 (6.2–10.4)	8.8 (6.8–11.1)	8.1 (6.2–10.2)
3 months (n)	134	130	125	90
Median (IQR)	8.3 (6.8–10)	8.3 (6.9–10.3)	8.3 (6.6–10)	8.4 (6.7–10.3)
6 months (n)	132	125	123	87
Median (IQR)	7.9 (6.5–9.6)	7.8 (6.3–9.2)	7.8 (6.4–9.5)	7.8 (6.2–9.2)
12 months (n)	130	127	121	88
Median (IQR)	7.2 (5.9–8.7)	7.6 (6.1–9.5)	7.2 (5.9–8.7)	7.8 (6.4–9.6)

NMP: normothermic machine perfusion; SCS: static cold storage.

Table 6. Assessment of normothermic machine perfusion (NMP) assessment score for the prediction of delayed graft function. Results are presented for patients who were transplanted and received NMP (there were 20 cases who were randomised to NMP but did not receive NMP and four who were not transplanted). Additionally, two participants did not have the assessment score populated. A logistic regression model was fitted, adjusting for cold ischaemic time, donor age, left/right kidney and centre (all as fixed effects) and with NMP assessment fitted as a binary variable, as there were few kidneys that scored ≥3.

NMP assessment score	**N = 117**	**%**	**Adjusted odds ratio (95% CI)**
1	63	54	
2	41	35	
3	10	9	
4	3	3	
5	0	0	
1	63	54	1.021 (0.466–2.237)
2 or more	54	46	1

CI: confidence interval.

Table 7. Sub-analysis of the primary outcome measure delayed graft function (DGF) including all primary non-function (PNF) cases. Nine patients were included in the modified intention-to-treat cohort and 6 in the per protocol cohort. PNF cases were treated as DGF. Odds ratio (OR) was calculated using a logistic regression model adjusted for cold ischaemic time, donor age, left/right kidney and transplant centre.
[1] P value obtained from the likelihood ratio test (two-sided test). Two participants did not have a cold ischemic time reported and two participants were transplanted as dual transplants from the same donor meaning that it was not possible to populate the left/right variable. Hence, these four cases were excluded from all risk adjusted modelling.

	**Modified intention-to-treat**		**Per protocol**	
Primary outcome	SCS (n=147)	NMP (n=143)	SCS (n=136)	NMP (n=96)
**DGF N (%)**	86/145 (59%)	88/141 (62%)	80/135 (59%)	57/95 (60%)
OR (95% CI)	1.18 (0.73–1.91)		1.11 (0.64–1.92)	
[1]P value	0.510		0.722	

Table 8. Post hoc subgroup analysis for delayed graft function modified intention-to-treat population for recipient pre-transplant dialysis status (on dialysis vs pre-dialysis). Patients who experienced primary non-function were excluded (n = 9). A logistic regression model was adjusted for cold ischaemic time, donor age, left/right kidney and centre, but with the inclusion of the pre-transplant dialysis status term and an interaction between treatment and pre-transplant dialysis status. The P value (two-sided) is from the likelihood ratio test when including and excluding the interaction term from the model.

Outcome	**On dialysis**		**Pre-dialysis**	
	SCS	NMP	SCS	NMP
**Delayed graft function n (%)**	76/113 (67%)	79/121 (65%)	9/31 (29%)	4/16 (25%)
OR (95% CI)	0.96 (0.55–1.66)		0.87 (0.21–3.55)	
P value	0.90			

CI: confidence interval; NMP: normothermic machine perfusion; OR: odds ratio; SCS: static cold storage.

Table 9. Number of transplanted patients with only 1 day of dialysis within the first 7 days post-transplant.

	**Modified intention-to-treat**		**Per protocol**	
	SCS (n=147)	NMP (n=143)	SCS (n=136)	NMP (n=96)
No. DGF	83	82	77	54
No. DGF patients with duration of DGF complete	76	70	70	47
No. DGF patients with duration of DGF = 1 day	13	16	13	14

DGF: delayed graft function; NMP: normothermic machine perfusion; SCS: static cold storage.

Table 10. Sub-analysis of the creatinine reduction ratio day 2 (CRR2) excluding patients not on dialysis pre-transplant. Nine patients were excluded from the modified intention-to-treat cohort and 6 from the per protocol cohort due to primary non-function (PNF). Mean ± SD are unadjusted Kaplan–Meier estimates. [2] P value obtained from the log-rank test (two-sided test).

	**Modified intention-to-treat**		**Per protocol**	
	SCS (n=147)	NMP (n=143)	SCS (n=136)	NMP (n=96)
**CRR2**				
n	37	42	36	30
Mean ± SD	0.90 ± 16.50	9.23 ± 17.03	0.95 ± 16.73	10.73 ± 13.44
Mean difference	8.31 (0.78–15.83)		10.63 (3.06–18.19)	
P value	0.0309		0.0067	

NMP: normothermic machine perfusion; SCS: static cold storage.

Table 11. Sub-analysis of the modified intention-to-treat analysis populations with imputed estimated glomerular filtration rate (eGFR) at 1, 3, 6 and 12 months post-transplant, in the static cold storage (SCS) and normothermic machine perfusion (NMP) arms of the trial. All missing estimated glomerular filtration rate (eGFR) values including those due to primary non-function (PNF), delayed graft function (DGF), patient death and graft loss were imputed with the value 8.5 mL/min/1.73m^2. All mean differences, P values and hazard ratios were adjusted for cold ischaemic time, donor age, left/right kidney indicator and centre. Mean values of eGFR are unadjusted and mean difference was calculated using adjusted normal linear regression model with random effect. [1] P value obtained from the likelihood ratio test (two-sided test).

	Modified intention-to-treat		Per protocol	
	SCS (n=147)	NMP (n=143)	NA	NA
**eGFR (mL/min/1.73m^2)**				
1 month (n)	147	143		
Mean ± SD	33 ± 16	33 ± 18		
3 months (n)	147	143		
Mean ± SD	38 ± 17	39 ± 19		
6 months (n)	147	143		
Mean ± SD	38 ± 18	39 ± 19		
12 months (n)	147	143		
Mean ± SD	41 ± 21	41 ± 20		
**Mean difference at**				
1 month	0.40 (–3.46–4.26)			
3 months	1.32 (–2.55–5.18)			
6 months	1.31 (–2.55–5.17)			
12 months	–0.61 (–4.47–3.25)			
[1]P value	0.59			

Table 12. The effect of the second period of cold ischaemia on rates of delayed graft function (DGF) in normothermic machine perfusion (NMP) kidneys in the modified intention-to-treat and per protocol analysis**.** Odds ratio (OR) reflects the increase in odds of DGF per minute of 'second period of cold ischaemia time'. [1] P value obtained from the likelihood ratio test (two-sided test).

	**Modified intention-to-treat**	**Per protocol**
	NMP (n=143)	NMP (n=96)
**Second period of cold ischaemia time**		
OR* (95% CI)	1.001 (0.998–1.004)	1.000 (0.997–1.004)
[1]P value	0.4451	0.8545

*Adjusted for donor age, left/right kidney and transplant centre.

Protocol

A Randomised controlled, open label trial of Ex-vivo Normothermic Perfusion versus Static Cold Storage in Donation after Circulatory Death Renal Transplantation

EVNP DCD Kidney Trial

IRAS Number: 179752

ISRCTN15821205

Protocol Version 1.4

Date: 5th May 2019

Sponsor: University of Cambridge and University Hospitals of Cambridge Foundation Trust

Funder: Kidney Research UK (SP/MEKC/1/2014)

**Principal Investigator**	Professor Michael Nicholson<br>Addenbrooke's Hospital, Level 9<br>Hill's Road, Cambridge CB2 OQQ<br>Tel 01223 762002
**Trial Manager &<br>Co-investigator**	Dr Sarah Hosgood<br>Addenbrooke's Hospital, Level 9<br>Hill's Road, Cambridge CB2 OQQ<br>Tel 01223 762002
**Lead Statistician &<br>Co-investigator**	Professor David Collett<br>NHS Blood and Transplant
**Trial Statistician**	Laura Pankhurst<br>Statistics and Clinical Studies<br>NHS Blood and Transplant<br>Tel: 0117 975 7431
**Data Manager**	Siobhan Martin<br>NHS Blood and Transplant CTU<br>Tel: 01223 588096
**Principal Investigator (Newcastle)**	Mr Colin Wilson<br>Freeman Hospital, Newcastle
**Principal Investigator (Guy's)**	Mr Chris Callaghan<br>Guy's & Thomas' NHS Foundation Trust
**Co-investigator**	Mr Kourosh Saeb-Parsy<br>Addenbrooke's Hospital, Cambridge
**Co-investigator**	Mr Gavin Pettigrew<br>Addenbrooke's Hospital, Cambridge
**Co-investigator**	Dr Nicholas Torpey<br>Addenbrooke's Hospital, Cambridge
**Co-investigator**	Professor Christopher Watson<br>Addenbrooke's Hospital, Cambridge
**Co-investigator**	Dr Eleanor Bolton<br>Addenbrooke's Hospital, Cambridge
**Co-investigator**	Professor J Andrew Bradley<br>Addenbrooke's Hospital, Cambridge
**Sponsor**	University of Cambridge and Cambridge University Hospitals NHS Foundation Trust' Addenbrookes Hospital Hill's Road, Cambridge CB2 OQQ. Mr

Stephen Kelleher Tel 01223 596472.
r&denquiries@addenbrookes.nhs.uk

List of Contents

1: Title page	Page 1
2: List of investigators	Page 2
3: List of abbreviations	Page 4
4: Study synopsis	Page 5
5: Flow chart	Page 7
6: Introduction	Page 8
7: Trial design	Page 9
8: Selection and withdrawal of patients	Page 10
9: Trial procedures	Page 11
10: Study outcome measures	Page 14
11: Assessment of safety	Page 15
12: Statistics	Page 16
13: Research Governance	Page 18
14: Responsibilities	Page 19
15: References	Page 21
16: Appendix 1	Page 22
17: Appendix 2	Page 25
18: Appendix 3	Page 26

List of Abbreviations

ATP	Adenosine triphosphate
CRF	Case report forms
CRR2	Creatinine reduction ratio day 2
CS	Cold storage
DCD	Donation after circulatory death
DGF	Delayed graft function
DMC	Data monitoring committee
eGFR	estimated glomerular filtration rate
EVNP	Ex-vivo normothermic perfusion
fDGF	functional delayed graft function
NHSBT	National Health Service Blood and Transplant
PNF	Primary non-function
RBF	Renal blood flow
RCT	Randomised controlled trial
SAE	Serious adverse event
TMC	Trial management committee
TSC	Trial steering committee
UO	Urine output

1. STUDY SYNOPSIS

Title of trial	Randomised controlled, open label trial of ex-vivo normothermic perfusion (EVNP) versus cold storage (CS) in DCD kidney transplantation
Aim of clinical trial	To determine if a short period of reconditioning using EVNP improves initial graft function in DCD kidney Transplants
Primary outcome measure	Delayed graft function (DGF) defined as the need for dialysis in the first 7 days post-transplantation
Secondary outcome measures	1. Primary non-function (PNF) defined as the permanent lack of allograft function from the time of transplantation. This will include graft losses due to irreversible rejection and vascular thrombosis. The cause of graft loss will be recorded.<br>2. Duration of DGF in days.<br>3. Functional DGF (fDGF) defined as <10% fall in serum creatinine for 3 consecutive days in the first week post-transplant.<br>4. Creatinine reduction ratio day 2 (CRR2 = creatinine day 1 – creatinine day 2/ creatinine day 1).<br>5. Creatinine reduction ratio day 5 (CRR 5 = Day 1 creatinine - creatinine day 5/ day 1 transplant creatinine).<br>6: Length of hospital stay<br>7. Biopsy-proven acute rejection rates.<br>8. Serum creatinine and eGFR at 1, 3, 6 and 12 months.<br>9. Patient survival (time from transplant to death).<br>10. Allograft survival (time from transplant to graft loss or return to dialysis)
Study design	Randomised, controlled, open-label clinical trial
Sample size	400 DCD kidney transplants
Eligibility criteria	Patients undergoing DCD kidney transplantation<br>Patients aged 18 years and above<br>First and second transplants
Investigational medicinal product	This is not a trial of an investigational medicine
Screening and enrolment	All patients undergoing DCD kidney transplantation<br>Will be considered for enrolment
Treatment period	The intervention is non-pharmacological<br>DCD kidneys will be randomised to undergo preservation by cold storage (CS) alone or CS followed by a one-hour

	period EVNP immediately prior to transplantation. This will be followed by kidney transplantation using standard techniques.
Follow up period	The main end-point will be at one year but patients will be followed-up for 5 years
Procedures for safety and Monitoring	Serious adverse event reports will be forwarded to the data monitoring committee. Non-serious adverse events will be recorded and reported to the data monitoring committee.

Flow Chart

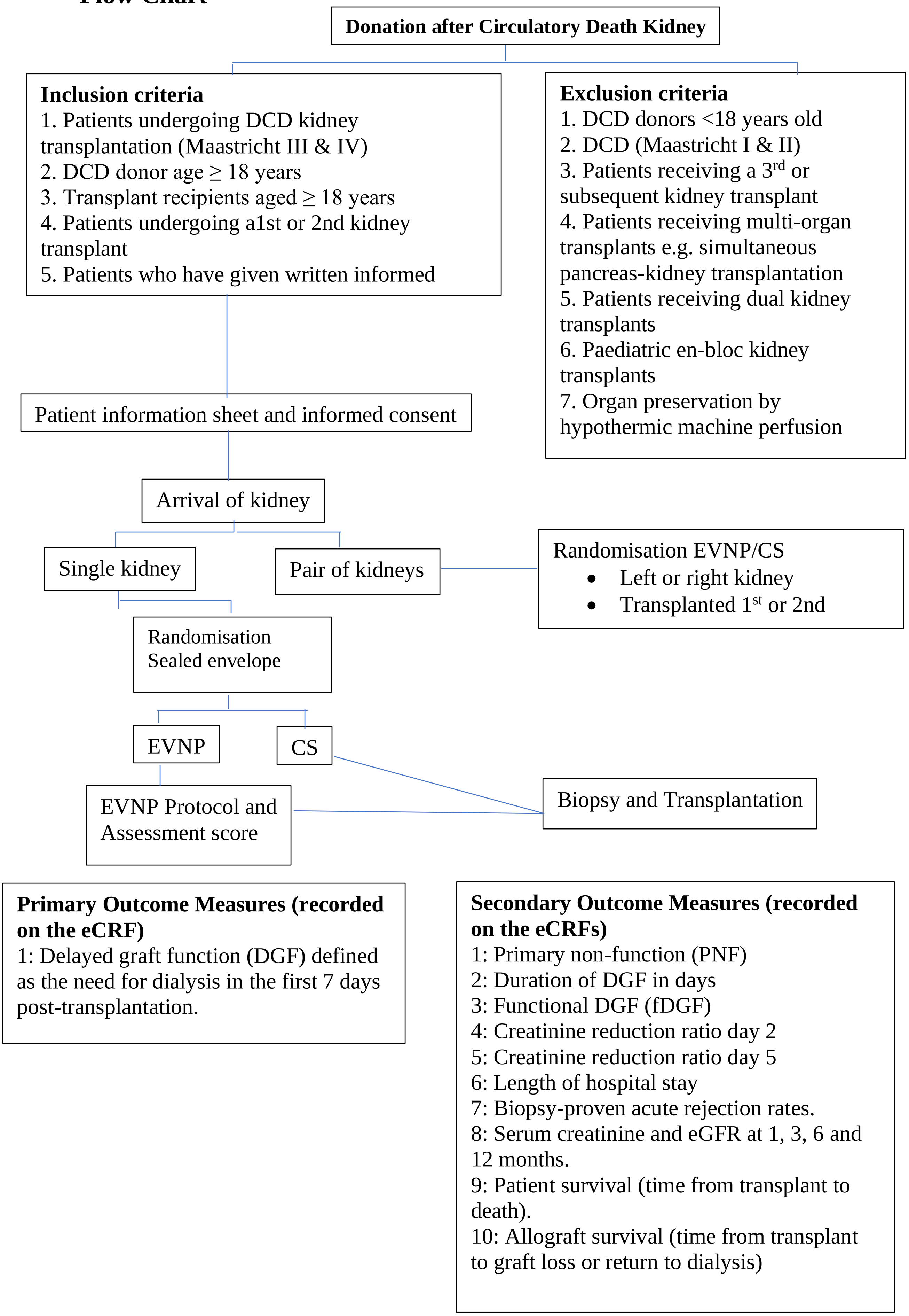
Donation after Circulatory Death Kidney
Inclusion criteria
1. Patients undergoing DCD kidney transplantation (Maastricht III & IV)
2. DCD donor age ≥ 18 years
3. Transplant recipients aged ≥ 18 years
4. Patients undergoing a1st or 2nd kidney transplant
5. Patients who have given written informed
Exclusion criteria
1. DCD donors <18 years old
2. DCD (Maastricht I & II)
3. Patients receiving a 3rd or subsequent kidney transplant
4. Patients receiving multi-organ transplants e.g. simultaneous pancreas-kidney transplantation
5. Patients receiving dual kidney transplants
6. Paediatric en-bloc kidney transplants
7. Organ preservation by hypothermic machine perfusion
Patient information sheet and informed consent
Arrival of kidney
Single kidney
Pair of kidneys
Randomisation EVNP/CS
Left or right kidney
Transplanted 1st or 2nd
Randomisation
Sealed envelope
EVNP
CS
EVNP Protocol and Assessment score
Biopsy and Transplantation
Primary Outcome Measures (recorded on the eCRF)
1: Delayed graft function (DGF) defined as the need for dialysis in the first 7 days post-transplantation.
Secondary Outcome Measures (recorded on the eCRFs)
1: Primary non-function (PNF)
2: Duration of DGF in days
3: Functional DGF (fDGF)
4: Creatinine reduction ratio day 2
5: Creatinine reduction ratio day 5
6: Length of hospital stay
7: Biopsy-proven acute rejection rates.
8: Serum creatinine and eGFR at 1, 3, 6 and 12 months.
9: Patient survival (time from transplant to death).
10: Allograft survival (time from transplant to graft loss or return to dialysis)

2. INTRODUCTION

2.1 Background

Kidney transplantation continues to be limited by a shortage of organ donors. In response to this there has been an increase in the use of kidneys from marginal donors and a significant proportion of transplant kidneys are now provided by donation after circulatory death (DCD). DCD kidneys inevitably sustain a warm ischaemic insult prior to retrieval and 50-60% of these kidneys have delayed graft function (DGF) after transplantation.

DGF occurs due to tubular cell damage (acute tubular necrosis, ATN). Release of damage-associated molecular patterns (DAMPs) from dying cells leads to activation of resident immune cells, including NLRP3 inflammasome assembly. This results in the release of pro-inflammatory cytokines, including IL1β and IL18, further exacerbating tissue damage. ATN is associated with an increased risk of acute rejection, longer in-patient stay and therefore greater cost, and may also reduce allograft survival (1-6). Moreover, when compared to standard criteria donors, DCD kidneys are three times more likely to be declined for transplantation due to concerns over their quality (7, 8).

Organ preservation has traditionally relied on hypothermic techniques based on the principle that refrigeration reduces cellular metabolism and tissue oxygen requirements. The problem is that in the anoxic hypothermic environment anaerobic cellular respiration continues, albeit at a slow pace. Oxidative phosphorylation is uncoupled and mitochondrial ATP production by chemiosmosis ceases. ATP continues to be generated at a much slower rate by substrate-based phosphorylation in the glycolytic pathway. This leads to depletion of intracellular ATP stores. Anaerobic respiration generates lactic acid leading to worsening intracellular acidosis and eventually loss of cell viability. The initial warm ischaemic injury sustained by DCD kidneys makes them less tolerant of cold ischaemic injury during hypothermic preservation.

Normothermic perfusion techniques offer an alternative form of organ preservation and resuscitation that has the potential to limit some of the effects of hypothermic preservation techniques. Ex-vivo normothermic perfusion (EVNP) is a novel technique that may help to recondition ischaemically injured kidneys prior to transplantation. The aim of EVNP is to restore metabolism and function to the kidney prior to transplantation by circulating a warm oxygenated red cell based solution through the kidney. This may also reduce subsequent immune activation following reperfusion.

EVNP has recently been introduced into clinical practice for kidneys from marginal donors (9-13). The early experience of renal transplantation after EVNP shows that the technique is feasible, safe and may improve early graft function.

2.2 Results of the first clinical series of renal transplants after EVNP

36 kidneys from marginal donors have been transplanted. The donor sources were: extended criteria donors (ECD; n=23), DCD (n=8) and standard criteria donors (SCD; n=5). The 5 SCD kidneys were considered as being marginal as they all had other adverse factors such as prolonged warm ischaemia. This series of 36 EVNP kidneys was compared to a contemporaneous group of 47 ECD kidneys transplanted after CS alone.

All 36 EVNP kidneys were perfused without any complications and all produced urine, although the amount varied significantly, ranging from 50 to 450mL. There was some fluctuation in the intra-renal resistance (IRR) during the first 15 minutes of EVNP in all kidneys but overall there was a general decline in IRR throughout perfusion.

There were no incidences of primary non-function (PNF) in the EVNP or CS series. There were no episodes of renal artery or vein thrombosis and no urological complications. The rate of

DGF, defined as the requirement for dialysis in the first 7 days, was 4/36 (11%) in the EVNP group versus 17/47 (35%) in the CS group; P=0.011 (Fisher's exact test). The incidence of acute rejection was similar in both groups (EVNP 6/36 [17%] vs. CS 9/47 [19%]; p = 0.753). There were no differences in graft or patient survival at 12 months (p = 0.510 and 1.000 respectively). The median length of follow up was 640 days (range 243–1521) in the CS kidneys and 356 days (range 30–640) in the EVNP kidneys.

3. TRIAL DESIGN

3.1 Statement of trial design

A randomised controlled, open label trial of the effect of EVNP on initial graft function in donation after circulatory death kidney transplantation.

3.2 Number of centres

Patients will be recruited from 6 centres:

1. Addenbrooke's Hospital, Cambridge
2. Freeman Hospital, Newcastle upon Tyne
3. Guy's and St Thomas's, London
4. Edinburgh Royal Infirmary
5. Royal London Hospital
6. Queen Elizabeth Hospital, Glasgow

3.3 Trial Management Group

The trial will be co-ordinated by a Trial Management Group (TMG) at Addenbrooke's Hospital. The members of the Trial Management group are as follows; (Michael Nicholson, Sarah Hosgood, Alison Deary, Laura Pankhurst and Siobhan Martin).

3.4 Number of participants

A total sample size of 400 participants will need to be recruited across the 6 centres.

3.5 Randomisation

Recruits will be allocated at random in a 1:1 ratio to either CS plus 1 hour of EVNP or CS only. A patient information sheet will be given to potential recruits on admission for DCD kidney transplantation and written informed consent will then be obtained. Randomisation will be performed after the transplant recipient and kidney have both arrived in the transplanting centre and a final decision to proceed with transplantation has been made. The randomisation will be performed by an independent company who will use a sealed envelope system created using a computer generated randomisation sequence. In cases where paired kidneys from the same donor are transplanted in the same centre, one kidney from the pair will be randomly allocated to CS and the other to EVNP. In these cases the randomisation will also determine which kidney (right or left) will be transplanted first.

3.5.1 Randomisation The Royal London

Kidneys accepted by The Royal London hospital will be randomised by Guy's & St Thomas's after written informed consent is taken.

RANDOMISATIONS
To randomise, please visit

www.sealedenvelope.com

Choose the EVNP trial from the drop down list and log-on as instructed

3.6 Estimated recruitment rate

Table 1 shows the number of DCD kidney transplants performed in the three recruitment centres over a three-year period (NHSBT data). A total of 550 DCD kidneys transplants were performed in the 3 centres during the period 2011-2014, with a mean of 183 transplants per year. The proposed trial requires 100 transplants per annum over a four-year period. Assuming that the recent DCD transplant rates are maintained then the required recruitment rate to enroll 400 patients is 55%. Recruitment may be decreased by the introduction of national organ sharing for DCD kidneys.

Table 1: DCD kidney Transplant Rates in the Three Trial Centres 2011-14

**Centre**	**2011-12**	**2012-13**	**2013-14**	**3 year Total**
Cambridge	68	99	78	245
Newcastle	49	57	51	157
Guy’s	16	63	69	148
Total	133	219	198	550

4. SELECTION AND WITHDRAWAL OF PATIENTS

4.1 Inclusion criteria

1. Patients undergoing DCD kidney transplantation (Maastricht Categories III & IV)
2. DCD donor age ≥ 18 years
3. Transplant recipients aged ≥ 18 years
4. Patients undergoing a1st or 2nd kidney transplant
5. Patients who have given written informed consent

4.2 Exclusion criteria

1. DCD donors <18 years old
2. DCD donors in Maastricht Categories I & II

3. Patients receiving a 3rd or subsequent kidney transplant
4. Patients receiving multi-organ transplants e.g. simultaneous pancreas-kidney transplantation
5. Patients receiving dual kidney transplants
6. Paediatric en-bloc kidney transplants
7. Organ preservation by hypothermic machine perfusion
8. One of a pair already randomised as a single kidney
9. Donor undergoes normothermic regional perfusion

4.3. Multiple renal arteries

DCD kidneys with multiple renal arteries will not be excluded from the trial as EVNP is technically possible in such kidneys. Nonetheless, EVNP may prove to be very difficult in kidneys with particularly complex vascular anatomy. If such a kidney is randomised to EVNP then the local investigator may decide to use CS alone. Nonetheless, the recipient will be analysed in the EVNP arm of the study in line with the intention to treat basis. As per protocol analysis will also be performed to assess the effects of the actual preservation method.

4.4 Patient withdrawal criteria

Once informed consent has been obtained, withdrawal from the study is likely to be uncommon.

4.4.1 Criteria for withdrawal from the study

- Withdrawal of consent by the patient.
- The kidney being deemed untransplantable following final bench surgery e.g irreparable vascular damage.

4.4.2 When and how to withdraw patients from the study

Withdrawal can happen at any time after gaining informed consent or during the peri and post-transplantation period. No specific procedures are required for withdrawal. The end of study case record form (CRF) must be completed with the reasons for withdrawal.

4.4.3 Follow up of withdrawn patients

Patients who are randomised but withdrawn before intervention will receive standard clinical care according to the local protocol. If patients undergo the intervention but subsequently withdraw they will also receive standard clinical care. In the unexpected situation where consent to use data and samples that have already been collected is withdrawn, then these will be discarded.

5.0 TRIAL PROCEDURES

5.1 Overview of trial procedures

After the identification of a suitable DCD kidney and matched recipient, the patient will be called into the transplant centre. Once assessed and the inclusion criteria met, the patient will be given the patient information sheet and the trial explained to them in full. Informed consented will be taken by a qualified member of the research team after the patient has been given an appropriate length of time to read the information sheet. Randomisation will be performed after the transplant recipient and kidney have both arrived in the transplanting centre and a final decision to proceed with transplantation has been made. The randomisation will be performed by an independent company who will use a sealed envelope system created using a computer generated randomisation sequence. A member of the transplant research team will access this. In cases where paired kidneys from the same donor are transplanted in the same centre, one kidney from the pair will be randomly allocated to CS and the other to EVNP. In these cases the

randomisation will also determine which kidney (right or left) will be transplanted first. If a kidney is randomized as a single and the other paired kidney is accepted by the centre at a later time, the second kidney will not be included in the trial.
During the transplant procedure a biopsy will be taken prior to transplantation then where possible 30 minutes after reperfusion. Routine daily blood samples will be collected from the patients to monitor kidney function during their hospital stay. Some samples will be stored and used to measure kidney injury. In a supplementary study of 30 patients additional samples will be collected to measure recovery and immune activation. After discharge patients will be monitored as normal during routine clinic visits. Patients will visit the transplant clinic or be followed up at their local centre at 1, 3, 6, and 12 months post-transplant. At 3 months post-transplants patients will be asked to undergo a renal transplant biopsy performed under local anaesthetic and ultrasound guidance. This is not mandatory. A trained member of staff will perform this on the day ward within the hospital. Renal function, and any complications will be recorded at each visit using the electronic case report forms. Patients will be followed up as normal practice for the duration of their functioning transplant kidney.

5.1.2 The Royal London

For eligible recipients recruited at The Royal London. The transplant team at Guys and the Royal London hospital will liaise closely to ensure that no delays are incurred. After written consent has been obtained the kidney will randomized by the Transplant team at Guy's & St Thomas's. Kidneys randomized to EVNP will be promptly transferred to Guy's & St Thomas's for perfusion. After EVNP, kidneys will be flushed and packed in ice and immediately transported by a courier to The Royal London for transplantation. The second period of cold ischaemia due to transportation will not alter the design of the study compared to kidneys remaining in the same centre. Kidneys that are transplanted within the same centre often undergo a necessary second period of cold ischaemic after EVNP due to logistics and access to theatre.

5.2 Kidney retrieval

All kidneys will be retrieved by UK national retrieval teams. Following *in situ* flushing of the abdominal organs with hyperosmolar citrate solution or University of Wisconsin (UW) solution, kidneys will be removed and then placed individually in preservation solution and packed in ice. The perfusion solution used will be recorded.

5.3 Ex-vivo normothermic perfusion (Appendix 3)

5.3.1 Perfusion Machine

Details of the EVNP procedure is detailed in appendix 3. The EVNP circuit has been designed using paediatric cardiopulmonary bypass technology (Medtronic, Watford, UK) and consists of a centrifugal blood pump (Bio-pump 560), a heat exchanger, a venous reservoir (Medtronic), 1/4 inch PVC tubing and an Affinity membrane oxygenator (Medtronic). The hardware includes a speed controller, a TX50P flow transducer and a temperature probe (Cole-Parmer, London, UK). Two infusion pumps are also incorporated into the system.

5.3.2 Perfusate

The circuit will be primed with perfusate solution (Ringer's solution, Baxter Healthcare, Thetford, Norfolk, UK) and one unit of either O positive or O negative packed red cells from the blood bank. Manntiol 10% (Baxter Healthcare), dexamethasone 8 mg (Organon Laboratories, Cambridge, UK) and heparin (CP Pharmaceuticals, Wrexham, UK) will be added to the

perfusate. Sodium bicarbonate 8.4% (Fresenius Kabi, Cheshire, UK) will be added to normalize the pH. A nutrient solution (Synthamin 17, Baxter Healthcare) with sodium bicarbonate 8.4%, insulin (Novo Nordisk, Denmark) and multivitamins (Cernevit, Baxter Healthcare) will be infused into the circuit at a rate of 20 mL/h. Prostacyclin 0.5 mg (Flolan, Glaxo-Wellcome, Middlesex, UK) will be infused into the arterial arm of the circuit at a rate of 5 mL/h and glucose 5% (Baxter Healthcare) at 5 mL/h. Ringer's solution will be used to replace urine output mL for ml.

5.3.3 Perfusion Chamber

Kidneys undergoing EVNP will be placed in a custom designed sterile perfusion chamber and the renal artery and vein will be cannulated and primed with cold 0.9% sodium chloride. Care must be taken to exclude any air from the circuit at the start of perfusion.

5.3.4 Perfusion Parameters

Kidneys will be perfused at a set mean arterial pressure (75 mmHg). The plasma-free red cell-based perfusate will be circulated from the venous reservoir through the centrifugal pump into the membrane oxygenator, where it is oxygenated and also warmed to 32–37°C. It will then flow through the arterial limb of the circuit to the renal artery. Venous return from the renal vein will be fed back into the reservoir (Appendix 3).

Renal blood flow (RBF) will be monitored continuously during EVNP. Intra-renal resistance (IRR) will be calculated (mean arterial pressure/RBF) every 5 min until the end of perfusion. The total urine output will be recorded. Blood gas analysis will be used to measure the acid base balance pre and post EVNP.

5.3.5 Post-perfusion

After EVNP, kidneys will be flushed with approximately 500 to 1000 mL of cold (4°C) hyperosmolar citrate (HOC, Baxter Healthcare, UK) to remove the perfusate and then placed back in ice until transplanted. Prior to transplantation the arterial Carrel patch may have to be excised along with a short segment of vein in order to remove the cannula ligature sites.

5.3.6 EVNP Assessment Scoring

An assessment score will be recorded for all kidneys undergoing EVNP but will not be used to make decisions about the suitability of kidneys for transplantation (13).

Method: A visual assessment of the macroscopic appearance of the kidney will be made throughout the 60 minutes of EVNP. In addition, the mean renal blood flow and total urine output will be measured over 60 minutes of EVNP. Scores will be attributed as follows:

Macroscopic perfusion:

Excellent perfusion with global and even pink appearance (1 point)

Moderate perfusion with some areas of patchy or mottled perfusion (2 points)

Poor perfusion with a globally mottled and purple appearance (3 points)

Renal blood flow (RBF)

RBF < 50 ml/hr/100g (1 additional point)

Urine output (UO)

UO < 43 ml/hr (1 additional point)

Scores for macroscopic appearance, renal blood flow and urine output will be added to yield an overall assessment score ranging from 1 (the highest quality) to 5 (the lowest quality).

5.4 Samples

Tissue samples from the transplant kidney, blood and urine samples from the transplant recipients and samples of the blood based perfusion solution and urine from the kidneys during EVNP will be collected. Samples will be collected by a trained member of staff.
Tissue samples of the transplant kidney will be assessed for routine histology and for the measurement of fibrosis. Where possible tissue will be collected pre-implantation, 30 minutes post-transplant and at 3 months post-transplant. Tissue samples will be kept within the histology or within the transplant department at the research sites. Where possible a section of the tissue sample will be frozen in liquid nitrogen or placed in RNAlater for processing. Tissue will either be stored at 4˚C or at -80˚C within the University/NHS departments.
Blood and urine samples from the recipient will be used for routine biochemistry and haematology. Where possible a sample of blood and urine will also be kept for analysis of injury markers. Samples will be collected from the patient pre transplant, 6h, 24h, 48h and 72h post-transplant.
Plasma and urine samples from the kidney during EVNP will also be kept to assess further injury markers.
All blood and urine samples used to assess injury markers will be kept on ice then centrifuged at 1600 RPM at 4˚C for 10 minutes. The supernatant will be collected and stored at -80˚C within the transplant departments.

5.4.1 Storage

The samples will be stored in the clinical and research laboratories within the participating centres. Samples will be coded and donor identifiable material will only be available to the PI and the research team at the participating centres. Samples will be stored within the department at the transplant centre then sent to the University of Cambridge for analysis.

It is the responsibility of the trial site to ensure that samples are appropriately labelled in accordance with the trial procedures to comply with the 1998 Data Protection Act. Biological samples collected from participants as part of this trial will be transported, stored, accessed and processed in accordance with national legislation relating to the use and storage of human tissue for research purposes and such activities shall at least meet the requirements as set out in the 2004 Human Tissue Act and the 2006 Human Tissue (Scotland) Act."

5.4.2 Disposal

On completion of the trial samples will be disposed of in accordance with the Human Tissue Authority's Code of Practice.

5.5 Transplantation

This will be performed using standard techniques. Kidneys can be transplanted into either iliac fossa with anastomosis of the artery to either the common, external or internal iliac arteries. The vein will be anastomosed to either the common or the external iliac vein. The ureteric anastomosis will be performed as an extravesical onlay over a double J stent.

5.6 Immunosuppressive therapy

All three centres will use similar immunosuppressive protocols as follows:

5.6.1 Basiliximab

Patients will receive 20mg of basiliximab i.v. pre-transplant and 20mg i.v. on postoperative day 4.

5.6.2 Prednisolone

Patients will receive a bolus of methylprednisolone i.v. at induction of anaesthesia using a dosage according to local practice. The post-transplant oral prednisolone regimen will also be according to local practice.

5.6.3 Calcineurin inhibitor

Patients may be treated with either tacrolimus or cyclosporine according to local protocols. There will be no restriction on the formulation of the prescribed calcineurin inhibitors. Tacrolimus will be prescribed to achieve target trough levels of 3-12 ng/ml. Cyclosporin will be prescribed to achieve target trough levels of 100-250 ng/ml. The first dose of calcineurin inhibitor may be given pre- or post-operatively.

5.6.4 Mycophenolate

All patients will receive mycophenolate as either mycophenolate mofetil (Cellcept) at a starting dose of at least 1g/day or mycophenolate sodium (Myfortic) starting at a dose of at least 720mg/day.

5.6.5 Campath

According to local protocols Campath may be used as an induction therapy as an alternative to Basiliximab.

5.7 Concomitant therapies

Anti-microbial and anti-thrombotic prophylaxis will be given according to local protocols. It is expected that patients will also receive prophylaxis against Pneumocystis jiroveci pneumonia, oral candidiasis and cytomegalovirus (Valganciclovir for 100 days in CMV +ve donor to CMV –ve recipient transplants).

5.8 Anaesthesia

This will be given according to local protocols.

6. STUDY OUTCOME MEASURES

6.1 Primary outcome measure

Delayed graft function (DGF) defined as the need for dialysis in the first 7 days post-transplantation.

6.2 Secondary outcome measures

1. Primary non-function (PNF) defined as the permanent lack of allograft function from the time of transplantation.. This will include graft losses due to irreversible rejection and vascular thrombosis. The cause of graft loss will be recorded.
2. Duration of DGF in days.
3. Functional DGF (fDGF) defined as <10% fall in serum creatinine for 3 consecutive days in the first week post-transplant.
4. Creatinine reduction ratio day 2 (CRR2 = creatinine day 1 – creatinine day 2/ creatinine day 1).
5. Creatinine reduction ratio day 5 (CRR 5 = Day 1 creatinine - creatinine day 5/ day 1 creatinine).
6. Length of hospital stay
7. Biopsy-proven acute rejection rates.
8. Serum creatinine and eGFR at 1, 3, 6 and 12 months.

9. Patient survival (time from transplant to death).
10. Allograft survival (time from transplant to graft loss or return to dialysis)

7. ASSESSMENTS OF SAFETY

7.1 Definitions

As this trial does not involve an investigational medicinal product all untoward incidents will be defined as adverse events rather than adverse reactions. Safety assessments will continue from the time of randomisation until completion of follow-up in all recipients.

7.1.1 Adverse Event

This is defined as any untoward medical occurrence affecting a patient without necessarily having any causality in relation to EVNP. The intensity of specific event can be categorized as mild, moderate or severe. An adverse event can be any unfavourable and unintended sign (including an abnormal laboratory finding), symptom, or disease temporally associated with the use of EVNP, whether or not considered related to this technique

7.1.2 Serious adverse event (SAE)

An adverse event is considered serious if it results in any of the following outcomes:
1. Results in death
2. Is life-threatening
3. Requires hospitalization or prolongation of existing in-patient hospitalization as a result of the intervention
4. Results in persistent or significant disability or incapacity

'Life-threatening' refers to an event in which the patient was at risk of death at the time of the event rather than an event, which hypothetically might have caused death if it were more severe.

7.1.3 Unexpected adverse event

This is defined as an adverse event, the nature or severity of which is not consistent with an expected consequence of EVNP.

7.2 Expected serious adverse events recognized to be caused by EVNP

There is a small risk of damage to the kidney vessels during preparation for EVNP. This risk is minimal (<1%).The early experience of EVNP suggests that it has an excellent safety profile and there are not expected to be any serious adverse events related to the perfusion period. If there are technical problems that lead to disconnection of the kidney from the circuit or there is poor perfusion during EVNP then the fail-safe position will be that the kidney can be quickly removed from the EVNP circuit, flushed with cold preservation solution and returned to CS.

7.3 Expected serious adverse events related to usual clinical care

These constitute the recognized complications of renal transplantation. They will not require completion of a SAE form but will be recorded in the Case Report Form. This will include the following complications:

1. Delayed graft function
2. Slow graft function
3. Acute rejection
4. Renal artery thrombosis
5. Renal vein thrombosis

6. Allograft loss for any reason
7. Urological complications, ureteric stenosis and /or urine leak
8. The complications of renal transplant biopsy
9. Admission for any other recognised complication of renal transplantation and immunosuppression

7.4 Reporting of unexpected serious adverse events

SAEs should be reported to the Trial Co-ordination Group within 7 days. The report should include an assessment of causality by the Principal Investigator. Notification of confirmed unexpected SAEs will be made to the Research Ethics Committee and the Data Monitoring Committee.

8. STATISTICAL CONSIDERATIONS

8.1 Method of Generating Allocation Sequence

Recruits will be randomised to CS plus 1 hour of EVNP or CS only, in a 1:1 allocation system. In cases where paired kidneys from the same donor are transplanted in the same centre, the randomisation will be stratified by kidney (right or left) so that one kidney is randomly allocated to each treatment and in which order they should be transplanted. Due to the nature of the trial, no-one is blinded to treatment allocation.

8.2 Outcome Measures

See section 6.

8.3 Sample size determination

The trial size was calculated with respect to the primary end-point, which is delayed graft function defined as the requirement for dialysis in the first 7 days post-transplantation. Based on 5 years of data from the three participating centres, the overall rate of DGF in DCD kidney transplants is 50%.

In a pilot series of kidney transplants from extended criteria donors, 18 kidneys undergoing CS followed by 1 hour of EVNP were compared to a historical control group of 47 ECD transplants after CS alone. The DGF rates were 1/18 (6%) in the EVNP group compared to 17/47 (36%) in the CS group (10).

Using a fixed sample size study, with interim analyses after 124 and 248 patients have been enrolled and reached 7 days post-transplant, a total of 370 patients receiving a DCD kidney (Maastricht category III & IV controlled donors) will be required to detect a 30% relative reduction in DGF (from 50 to 35%) with a power of 80% and a statistical significance of $\alpha = 0.05$. To allow for study withdrawal rate of 7.5%, a total of 400 patients will be recruited.

8.4 Interim Analysis

A group sequential design has been used to allow the Data Monitoring Committee to review the primary outcome for evidence of harm, benefit or futility. Two interim analyses will be performed during the study period. The first of these will be after 124 patients have been randomised and reached 7 days post-randomisation and the second after 248 patients have been randomised and reached 7 days post-randomisation. At these points, O'Brien-Fleming stopping rules for harm, benefit or futility will be used to guide the DMC review of the primary outcome data. The stopping rules will be used as a guideline, alongside the other safety data available to the DMC, and used as part of their overall assessment of the trial. They will have overall oversight and can recommend terminating the trial early for these or any other safety concerns. An overall significance level of 5% will be preserved at the end of the trial due to the use of the

O'Brien-Fleming stopping boundaries. The interim analysis will be unadjusted.

8.5 Analysis population and principles

The population used for efficacy analyses will be a modified intention to treat population including all randomised patients that received a transplant. This is a change from the original protocol because it was deemed illogical to include those participants who have not been transplanted as no outcome data will be available. This will be the primary analysis for the trial. Characteristics of all randomised patients will be tabulated by arm of the trial to describe the cohort.

The primary and secondary outcomes will also be analysed per protocol.

8.6 Analysis Plan (Brief)

8.6.1 Analysis of primary and secondary outcomes

The analyses will be described in detail in a full Statistical Analysis Plan. This section summarises the main issues. A full statistical analysis plan will be drawn up prior to the first interim analysis.

Primary analysis

The number of patients whom experience delayed graft function between the two groups will be presented and compared using a logistic regression model adjusting for cold ischemic time, donor age, left/right kidney and centre.

Secondary outcomes

- Primary non-function: The number of patients experiencing primary non-function in each arm will be compared using a logistic regression model adjusting for cold ischaemic time, donor age, left/right kidney and centre. The cause of graft loss will be tabulated by arm.
- Duration of DGF in days: The unadjusted median (and interquartile range) will be summarised using the Kaplan Meier estimate of the duration of DGF for all those who experienced DGF. Difference in duration of DGF will be assessed through the use of a Cox Proportional Hazards Regression model, adjusting for cold ischaemic time, donor age, left/right kidney indicator and centre.
- Number of participants whom experience functional DGF: The number of patients in each arm will be compared using a logistic regression model adjusting for cold ischaemic time, donor age, left/right kidney and centre.
- Creatinine reduction ratio day 2 (CRR2= (creatinine day 1 – creatinine day 2)/creatinine day 1): this ratio will be compared using a normal linear regression model adjusting for cold ischaemic time, donor age, left/right kidney and centre
- Creatinine reduction ratio day 5 (CRR5= (Creatinine day 1 – creatinine day 5)/creatinine day 1): this ratio will be compared a normal linear regression model adjusting for cold ischaemic time, donor age, left/right kidney and centre.
- Length of hospital stay: The unadjusted median (and interquartile range) time from admission to discharge will be obtained using the Kaplan Meier estimate. Difference in duration of hospital stay will be assessed through the use of a Cox Proportional Hazards Regression model, adjusting for cold ischaemic time, donor age, left/right kidney indicator and centre.

- Biopsy-proven acute rejection rate: The number of biopsy-proven rejection episodes per participant will be compared between treatment arms using a negative binomial model adjusted for cold ischaemic time, donor age, left/right kidney and centre
- Serum creatinine and eGFR at 1, 3, 6 and 12 months: longitudinal changes in serum creatinine and eGFR will be separately assessed at 1, 3, 6 and 12 months post-transplant. To assess if there is a difference in serum creatine (and separately eGFR) between the two arms over all time points, a normal linear regression model will be fitted adjusting for cold ischaemic time, donor age, left/right kidney, centre, time of measurement and a time by treatment interaction term (all as fixed effects). A random effect will also be included to allow for multiple measures per recipient.
- Patient survival (time from transplant to death): A Kaplan-Meier survival plot will be presented to show the unadjusted probability of patient death after 12 months by treatment arm. Difference in patient survival will be assessed using a Cox Proportional Hazards Regression model, adjusting for cold ischaemic time, donor age, left/right kidney indicator and centre,
- Allograft survival (time from transplant to graft loss or return to dialysis): Time to graft loss or return to dialysis will be modelled. Deaths will be censored, if the participant experienced death before the graft failure. A Kaplan-Meier survival plot will be presented to show the unadjusted probability of graft failure after 12 months by arm. Difference in graft survival will be assessed using a Cox Proportional Hazards Regression model, adjusting for cold ischaemic time, donor age, left/right kidney indicator and centre,

Safety outcomes
The total number of incidences of the following complications will be tabulated by arm:

1. Acute rejection
2. Renal artery thrombosis
3. Renal vein thrombosis
4. The complications of renal transplant biopsy
5. Number of hospital admission for any recognised complication of renal transplantation and immunosuppression

The number of each complication per participant will be summarised as an unadjusted mean and standard deviation by treatment arm. The number of each complication per participant will be compared between treatment arms using a Negative Binomial model adjusted for cold ischaemic time, donor age, left/right kidney and centre.

Other outcomes
1: EVNP assessment score: For those participants who received EVNP, the score will be tabulated with numbers and percentages. To assess for associations between DGF and the EVNP assessment score a logistic regression model will be fitted, adjusting for cold ischaemic time, donor age, left/right kidney and centre.

8.6.2 Missing Data and sensitivity analyses

Any missing primary and secondary outcome data will be summarised. Primary and secondary outcome measures will not be imputed and these will be treated as missing data and excluded from the relevant analyses. If outcome data is missing for more than 25% of participants, outcomes will not be reported.

To explore if missing values have an undue impact on the primary outcome result, a sensitivity analysis using multiple imputation will be performed if delayed graft function is missing in more

than 2% of the participants included in the modified intention to treat analysis. If the proportion of participants with a missing delayed graft function outcome is less than or equal to 2% then this sensitivity analysis will not be performed.

To explore if there was any effect of Royal London participants being randomised and undergoing EVNP at Guy's & St Thomas's, a sensitivity analysis will be conducted. The primary outcome will replicated excluding any participants from the Royal London.

8.6.3 Ancillary Studies

1: Renal fibrosis
One of the commonest causes for graft failure after transplantation is the development of chronic allograft nephropathy.
The aim of this study is to determine if EVNP can slow the progression of fibrosis. Biopsies of the kidney will be taken pre-transplant and where possible after 3 months.
Biopsies will be fixed in formalin and paraffin embedded cut sections of the graft with be stained with sirius red (stains for collagen III). The degree of fibrosis will be quantified using computerised digital image analysis.

2: Injury markers
Ischaemia reperfusion injury is a leading cause of early graft dysfunction. The aim of this study is to determine if EVNP can reduce the amount inflammation and injury after transplantation. Blood and urine samples will be collected pre and post-transplant and used to measure kidney injury and inflammation.

3: A supplementary observational cohort study of the immune response to acute kidney injury in renal transplant recipients.

In a subset of subjects recruited to this study (n=15 cold storage, n=15 EVNP), we will collect samples for analysis of immune activation, including biomarkers, flow cytometric analysis, and transcriptomic studies.

Sample Processing / Storage

Blood and urine samples will be taken alongside the main study samples, with additional sampling time points at 4-8 hours, day 1 and 2 post-transplant, at discharge and at 1, 3 6, 9 and 12 months. Samples will be collected from patients on the ward or clinic at Addenbrooke's Hospital and then transferred to the University of Cambridge or GlaxoSmithKline for storage and analysis to determine how EVNP affects immune activation.

Blood and urine samples from the recipient will be used to measure immune activation.

Blood: Peripheral blood will be collected for flow phenotyping in vitro stimulation assays and transcriptomic analysis.

Urine: Will be processed for immune activation markers.

Any data generated will be freely available to GSK and to the ENVP study chief and co-investigator (Prof Mike Nicholson and Dr Sarah Hosgood respectively). Any publications arising from data generated from these samples will be reviewed by all parties prior to submission.

9. RESEARCH GOVERNANCE

9.1 Trial Steering Committee

The University of Cambridge will be the sponsor for this study. The Trial Steering Committee will consist of: 3 independent experts, trial investigators (Lorna Marson, Grant Stewart and a lay member). It will meet 6 monthly to review the trial, monitor recruitment rates to consider protocol amendments and provide advice.

9.2 The Trial Management Group

The Trial Management Group (TMG) (Michael Nicholson, Sarah Hosgood, Alison Deary, Laura Pankhurst and Siobhan Martin) will be responsible for the ethics committee application and the production of reports and the day to day running of the trial. The committee will meet every 2 months or sooner if necessary.

9.3 Data Monitoring Committee

This will consist of three independent members with expertise in renal transplantation, clinical trials and statistics. The DMC will meet at least annually to review data, primarily concentrating on patient safety. The members of the committee are as follows;
Professor Paul White
Professor Peter Friend
Dr Chris Dudley

9.4 Data Handling

Data will be collected prospectively and recorded on the electronic CRFs. The data will be monitored by the trial manager and audited each month. Each participant will be allocated a unique study number and will be identifiable by this number throughout the course of the study. This number will be used on all documentation and during analysis of the results. Any data that is transferred will be carried out under the NHS Code of Practice on Confidentiality.

9.5 Ethical Considerations

- Before the start of the trial, approval will be sought from a REC for the trial protocol, informed consent forms and other relevant documents e.g. advertisements and GP information letters
- Substantial amendments that require review by REC will not be implemented until the REC grants a favourable opinion for the study (note that amendments may also need to be reviewed and accepted by the NHS R&D departments before they can be implemented in practice at sites)
- All correspondence with the REC will be retained in the Trial Master File/Investigator Site File

- An annual progress report (APR) will be submitted to the REC within 30 days of the anniversary date on which the favourable opinion was given, and annually until the trial is declared ended
- It is the Chief Investigator's responsibility to produce the annual reports as required.
- The Chief Investigator will notify the REC of the end of the study
- If the study is ended prematurely, the Chief Investigator will notify the REC, including the reasons for the premature termination
- Within one year after the end of the study, the Chief Investigator will submit a final report with the results, including any publications/abstracts, to the REC

9.6 Indemnity
The University insurance manager has advised that for insurance of negligent or non-negligent harm under the University's clinical trial policy can be arranged if this trial is approved by the NHS ethical committee. The University's insurers are Newline, the insurance policy reference is B0823Q31000177 and the limit of indemnity under the policy is £10m.

9.7 Dissemination
The results of the study will be submitted for peer review for publication in a scientific journal. The results of the study will also be presented at national and international meetings.

9.8 Amendments
The CI will notified the REC and NHS R&D departments of participating sites of any substantial amendments. Non-substantial amendments will be notified to the NHS R&D departments. Each amendment will be recorded in the investigator folder.

10. RESPONSIBILITIES

Principal Investigator (PI)
Checking for AEs when participants attend for treatment / follow-up.
1. Using medical judgement in assigning seriousness and causality and expectedness of SAEs.
2. Ensuring that all SAEs are recorded and reported to the Sponsor within 24 hours of becoming aware of the event and provide further follow-up information as soon as available. Ensuring that SAEs are chased with Sponsor if a record of receipt is not received within 2 working days of initial reporting.
3. Ensuring that AEs are recorded and reported to the Sponsor in line with the requirements of the protocol.

Chief Investigator (CI) / delegate or independent clinical reviewer
1. Clinical oversight of the safety of patients participating in the trial, including an ongoing review of the risk / benefit.
2. Using medical judgement in assigning seriousness, causality and expectedness of SAEs where it has not been possible to obtain local medical assessment.
3. Review of specific SAEs in accordance with the protocol.

Sponsor / delegate

1. Central data collection and verification of SAEs.
2. Reporting safety information to the CI, delegate or independent clinical reviewer for the ongoing assessment of the risk / benefit according to the Trial Monitoring Plan.
3. Reporting safety information to the independent oversight committees identified for the trial (Data Monitoring Committee (DMC) and / or Trial Steering Committee (TSC)) according to the Trial Monitoring Plan.
4. Checking for (annually) and notifying PIs of updates to the Reference Safety Information for the trial.

Trial Steering Committee (TSC)
In accordance with the Trial Terms of Reference for the TSC, periodically reviewing safety data and liaising with the DMC regarding safety issues.

Data Monitoring Committee (DMC)
In accordance with the Trial Terms of Reference for the DMC, periodically reviewing overall safety data to determine patterns and trends of events, or to identify safety issues, which would not be apparent on an individual case basis.

9. REFERENCES

1. Saidi RF, Elias N, Kawai T, et al. Outcome of kidney transplan- tation using expanded criteria donors and donation after cardiac death kidneys: Realities and costs. Am J Transplant 2007; 7: 2769–2774.
2. van der Vliet JA, Warle MC, Cheung CL, Teerenstra S, Hoitsma AJ. Influence of prolonged cold ischemia in renal transplantation. Clin Transplant 2011; 25: E612–E616.
3. Snoeijs MG, Winkens B, Heemskerk MB, et al. Kidney transplanta- tion from donors after cardiac death: A 25-year experience. Trans- plantation 2010; 90: 1106–1112.
4. Yarlagadda SG, Coca SG, Formica RN, Jr, Poggio ED, Parikh CR. Association between delayed graft function and allograft and pa- tient survival: A systematic review and meta-analysis. Nephrol Dial Transplant 2009; 24: 1039–1047.
5. Rao PS, Ojo A. The alphabet soup of kidney transplantation: SCD, DCD, ECD–fundamentals for the practicing nephrologist. Clin J Am Soc Nephrol 2009; 4: 1827–1831.
6. Quiroga I, McShane P, Koo DD, et al. Major effects of delayed graft function and cold ischaemia time on renal allograft survival. Nephrol Dial Transplant 2006; 21: 1689–1696.
7. Dare AJ, Pettigrew GJ, Saeb-Parsy K. Preoperative assessment of the deceased-donor kidney: from macroscopic appearance to molecular biomarkers. Transplantation 2014 Apr 27;97(8):797-807.
8. Callaghan CJ, Harper SJ, Saeb-Parsy K, Hudson A, Gibbs P, Watson CJ, et al. The discard of deceased donor kidneys in the UK. Clin Transplant 2014 Mar;28(3):345-353.
9. Hosgood SA, Nicholson ML. First in Man Renal Transplantation After Ex Vivo Normothermic Perfusion. Transplantation 2011 Aug 11.

10. Nicholson ML, Hosgood SA. Renal transplantation after ex vivo normothermic perfusion: the first clinical study. Am J Transplant 2013 May;13(5):1246-1252.
11. Hosgood SA, Nicholson ML. The first clinical case of intermediate ex vivo normothermic perfusion in renal transplantation. Am J Transplant 2014 Jul;14(7):1690-1692.
12. Hosgood SA, Nicholson ML. Ex vivo normothermic perfusion of declined human kidneys after inadequate in situ perfusion. Am J Transplant 2014 Feb;14(2):490-491.
13. Hosgood SA, Barlow AD, Hunter JP, Nicholson ML. Ex-vivo normothermic perfusion - an innovative technology for quality assessment of marginal donor kidney transplants. BJS. 2015 (in press)

APPENDIX 1

1. Risk

There is a small risk of damage to the kidney vessels during preparation for EVNP. This risk is minimal (<1%).The early experience of EVNP suggests that it has an excellent safety profile and there are not expected to be any serious adverse events related to the perfusion period. If there are technical problems that lead to disconnection of the kidney from the circuit or there is poor perfusion during EVNP then the fail-safe position will be that the kidney can be quickly removed from the EVNP circuit, flushed with cold preservation solution and returned to CS.

2. Study management/responsibilities
Study Sites

1: University of Cambridge
Addenbrooke's Hospital
Level 9, PO BOX 202
Hill's Road, Cambridge
CB2 OQQ
UK
PI/CI: Professor Michael Nicholson
Email: mln31@cam.ac.uk

2: The Newcastle Upon Tyne Hospitals NHS Foundation Trust
Freemans Hospital, Newcastle
Freemans Road,
High Heaton
Newcastle Upon Tyne and Wear
NE7 7DN
UK
CI: Mr Colin Wilson
Email: Colin.Wilson@nuth.nhs.uk

3: Guy's and St Thomas's NHS Foundation Trust
Trust Offices, Guy's Hospital
Great Maze Pond
London
Greater London
SE1 9RT
UK
CI: Mr Christopher Callaghan
Email: Chris.Callaghan@gstt.nhs.uk

2.1 Data Monitoring Committee
Contact details

Professor Paul White
Associate Professor (Statistics)
Applied Statistics Group
UWE Bristol
BS16 1QY
Email: Paul.White@uwe.ac.uk

Professor Peter Friend
University of Oxford
Nuffield Department of Surgical Sciences
Email: peter.friend@nds.ox.ac.uk

Dr Chris Dudley
Clinical Director of Renal & Transplant Directorate
North Bristol NHS Trust
Email: chris.dudley@nbt.nhs.uk

2.2 Trial Steering Committee
Contact details

Miss Lorna Marson
Senior Lecturer and Honorary Consultant Transplant Surgeon
Royal Infirmary Edinburgh
Tel: 0131 2421715
Email: Lorna.Marson@ed.ac.uk

Mr Grant Stewart
University of Cambridge
University Lecturer; Honorary Consultant Urological Surgeon
Addenbrooke's Hospital
Hill's Road
Cambridge CB2 OQQ
Email: gds35@cam.ac.uk

3: Patient registration
Patients recruited into the trial will be registered with The Clinical Trials Unit at NHSBT: Siobhan.Martin@nhsbt.nhs.uk. Tel: *01223 588096 (48096)*

4: Data management
The research teams at each site will be responsible for the completion and checking of the eCRFs. The Clinical Trials Unit will clarify any queries. Siobhan.Martin@nhsbt.nhs.uk.

5: Preparation and submission of amendments

The CI at the primary site will be responsible for the submission of any substantial amendments. The CI at participating sites (Newcastle and Guy’s) will be responsible for the submission of non-substantial amendments at their corresponding sites.

6: Preparation and submission of Annual Safety Report

The CI at the prinpicle site will be responsible for the preparation and submission of the annual safety report.

7: Trial documentation and archiving

The Clinical Trials Unit will be responsible for archiving the trial data.

8: Authorisation of participating sites

Required documentation

- Investigators CV
- Investigators GCP certificate
- R&D approval
- Site delegation log
- Site training log

NHS Blood and Transplant

Statistical Analysis Plan

EVNP DCD Kidney Trial

A Randomised controlled, open label trial of Ex-vivo Normothermic Perfusion versus Static Cold Storage in Donation after Circulatory Death Renal Transplantation

Kidney Research UK
Funding research to save lives

Version: Final 1.2

Date: 16/08/2021

CTU Ref: 15/85
ISRCTN: 15821205
REC: 15/EE/0356

Prepared by:

Name	Role	Signature	Date
Laura Smith	Trial Statistician		

Approved by:

Name	Role	Signature	Date
Professor Michael Nicholson	Chief Investigator		
Professor Lorna Marson	TSC Chair		
Cara Hudson	Lead Statistician		

1. Background and Design

The main characteristics of this trial have been summarised using the EVNP DCD Kidney Trial Protocol v1.4 from 5th May 2019. Please refer to this Protocol for full details.

1.1 Trial Summary objective

To determine if a short period of reconditioning using Ex-vivo Normothermic Perfusion (EVNP) improves initial graft function in DCD kidney transplants.

1.2 Patient Eligibility Criteria

Inclusion criteria

1. Patients undergoing DCD kidney transplantation (Maastricht Categories III & IV)
2. DCD donor age ≥ 18 years
3. Transplant recipients aged ≥ 18 years
4. Patients undergoing a 1st or 2nd kidney transplant
5. Patients who have given written informed consent

Exclusion criteria

1. DCD donors <18 years old
2. DCD donors in Maastricht Categories I & II
3. Patients receiving a 3rd or subsequent kidney transplant
4. Patients receiving multi-organ transplants e.g. simultaneous pancreas-kidney transplantation
5. Patients receiving dual kidney transplants
6. Paediatric en-bloc kidney transplants
7. Organ preservation by hypothermic machine perfusion
8. One of a pair already randomised as a single kidney
9. Donor undergoes normothermic regional perfusion

1.3 Trial Intervention

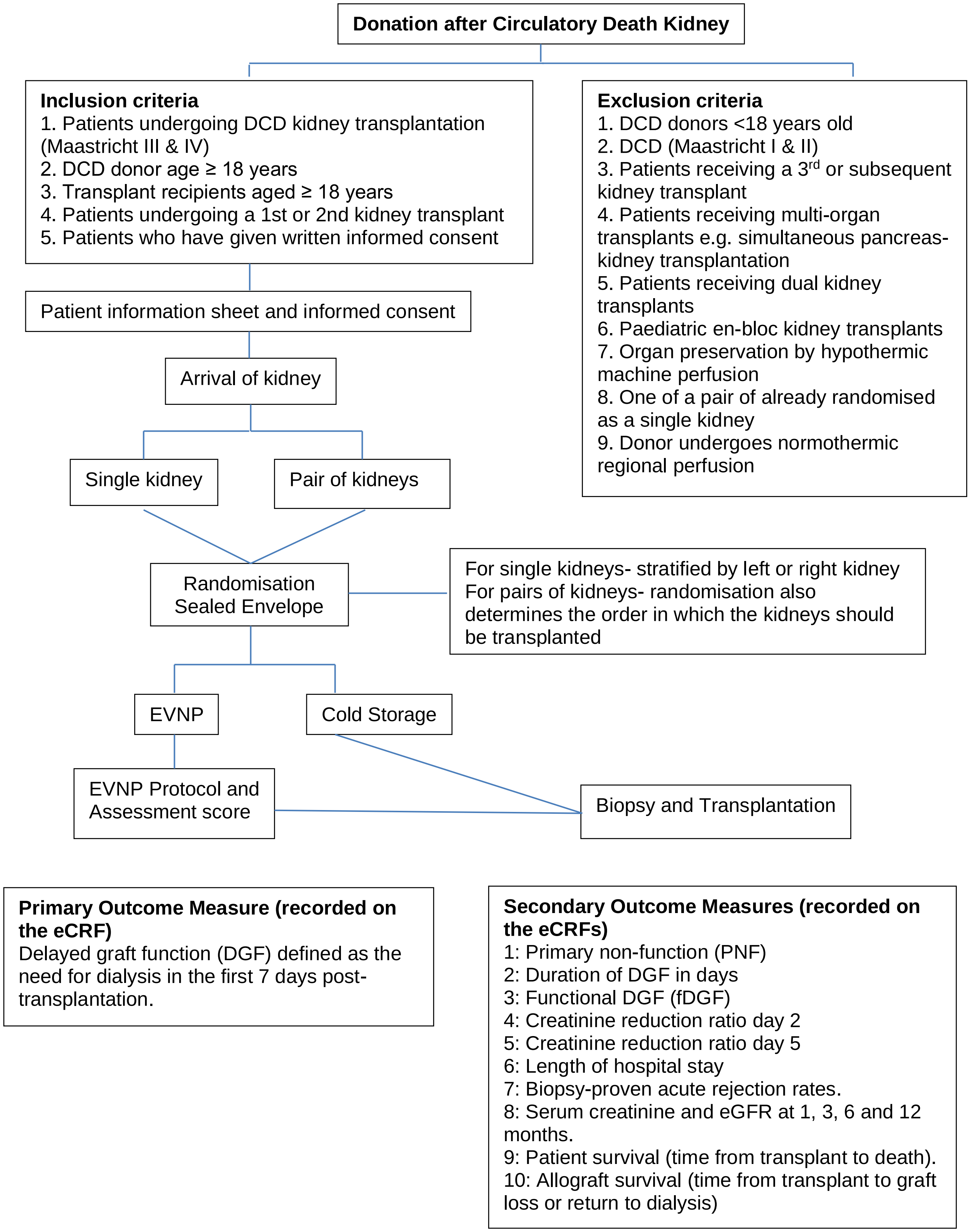

1.4 Randomisation and Blinding Procedures

Participants will be allocated at random in a 1:1 ratio to either Cold Storage (CS) plus 1 hour of EVNP or CS only. Randomisation will be performed after the transplant recipient

and kidney have both arrived in the transplanting centre and a final decision to proceed with transplantation has been made. The randomisation will be performed by an independent company using a computer generated randomisation sequence. In cases where paired kidneys from the same donor are transplanted in the same centre, one kidney from the pair will be randomly allocated to CS and the other to EVNP. In these cases the randomisation will also determine which kidney (right or left) will be transplanted first. For single kidneys the randomisation list was stratified by whether the kidney was left or right. No-one within the trial is blinded to treatment allocation.

According to the protocol, kidneys accepted by The Royal London hospital will be randomised by Guy's & St Thomas's after written informed consent is taken. However, this did not happen as the Royal London did not open to recruitment. Glasgow also did not open to recruitment.

1.5 Sample size calculation

The trial size was calculated with respect to the primary end-point, which is delayed graft function defined as the requirement for dialysis in the first 7 days post-transplantation. Based on 5 years of data from the three participating centres, the overall rate of Delayed Graft Function (DGF) in DCD kidney transplants is 50%.

In a pilot series of kidney transplants from extended criteria donors (ECD), 18 kidneys undergoing CS followed by 1 hour of EVNP were compared to a historical control group of 47 ECD transplants after CS alone. The DGF rates were 1/18 (6%) in the EVNP group compared to 17/47 (36%) in the CS group (10).

Using a fixed sample size study, with interim analyses after 124 and 248 participants have been enrolled and reached 7 days post-transplant, a total of 370 participants receiving a DCD kidney (Maastricht category III & IV controlled donors) will be required to detect a 30% relative reduction in DGF (from 50 to 35%) with a power of 80% and a statistical significance of $\alpha = 0.05$. To allow for study withdrawal rate of 7.5%, a total of 400 participants will be recruited.

2. Outcome Measures

2.1 Primary outcome measures

Delayed graft function (DGF) defined as the need for dialysis in the first 7 days post-transplantation.

2.2 Secondary outcome measures

- Primary non-function (PNF) defined as the permanent lack of allograft function from the time of transplantation. This will include graft losses due to irreversible rejection and vascular thrombosis. The cause of graft loss will be recorded.
- Duration of DGF in days.
- Functional DGF (fDGF) defined as <10% fall in serum creatinine for 3 consecutive days in the first week post-transplant.
- Creatinine reduction ratio day 2 (CRR2 = (creatinine day 1 – creatinine day 2)/ creatinine day 1).
- Creatinine reduction ratio day 5 (CRR 5 = (creatinine day 1 - creatinine day 5)/ creatinine day 1).
- Length of hospital stay
- Biopsy-proven acute rejection rates.
- Serum creatinine and eGFR at 1, 3, 6 and 12 months.
- Patient survival (time from transplant to death)

- Allograft survival (time from transplant to graft loss or return to dialysis)

3. Data Handling

3.1 CRF descriptions and data collection schedule

CRF			Completion requirement
Form no.	Page no.	Description of form	
1	2	Eligibility: Inclusion & Exclusion Criteria & Consent	All potentially eligible patients
2	3	Background Information: DONOR	All consented participants
3	4	Background Information: RECIPIENT	All consented participants
4	5	Pre-Transplant Therapy	All consented participants
5	6	Randomisation	All consented participants
6	7 & 8	Renal Anatomy and Bench Surgery	All consented participants
7	9 & 10	Ex-Vivo Normothermic (EVNP) Parameters	Participants randomised to EVNP
8	11	Intra-Operative Parameters	All transplanted participants
9	12	Biopsy, Blood & Urine Collection	All transplanted participants
10	13	Delayed Graft Function (DGF) & Primary Non Function (PNF)	All transplanted participants
11	14	Post Transplant: Functional DGF (fDGF) & Creatinine Reduction Ratio (CRR)	All transplanted participants
12	15	Post Transplant – 1 month (30 days ± 3 days)	All transplanted participants
13	16	Post Transplant – 3 months (90 days ± 7 days)	Participants who reached 1 month follow up
14	17	Post Transplant – 6 months (± 7 days)	Participants who reached 3 month follow up
15	18	Post Transplant – 12 months (± 14 days)	Participants who reached 6 month follow up
16	19	Post Transplant Therapy	All transplanted participants
17	20	Acute Rejection	If acute rejection is experienced
18	21	Complications of Renal Transplantation	If participant experiences a complication
19	22,23 & 24	Adverse Event reporting	If participant experiences an adverse event
20	25 & 26	Discontinuation, Hospital Discharge & Immunosuppressive drug type	All consented participants
21	27	End of Study Period	All transplanted participants

Trial Assessment schedule

Trial Assessment	Screening	Pre-Tx Therapy	Randomisation	DGF & PNF	Post Tx 1 month	Post Tx 3 months	Post Tx 6 months	Post Tx 12 months	Post-Tx Therapy	Discontinuation , Hospital Discharge & Acute rejection	End of Study Period	Acute Rejection	Complications of Renal Tx	Adverse Events
Eligibility	√													
Background Information - DONOR	√													
Background information - RECIPIENT	√													
Pre-Tx Therapy		√												
Randomisation			√											
Renal Anatomy & Bench Surgery			√											
EVNP Parameters			√											
Intra-Operative Parameters			√											
Biopsy, Blood & Urine Collection			√											
DGF & PNF				√										
Post Tx fDGF & CRR				√										
Post Tx– 1 month					√									
Post Tx – 3 months						√								
Post Tx – 6 months							√							
Post Tx– 12 months								√						
Post-Tx Therapy									√					
Discontinuation & Hospital Discharge										√				
End of Study Period											√			
Acute Rejection												√		
Complications of Renal Tx													√	
Adverse Events														√
Note: Tx = Transplant														

3.2 Procedures for recording and reporting outcomes

Prime responsibility for the complete collection of data for each centre will reside with the local Principal Investigator but may be delegated (for example to a research nurse). Overall responsibility for collating data from all centres will reside with the Trial Manager.

3.3 End-point Review Panel assessments

None

3.4 Other assessments

None

3.5 Trial Data Management and Verification

Quality control of data entered and data cleaning will be performed by the trial data manager and will be detailed in the Data Management Plan (FRM4727). This will include performing range, data completeness and consistency checks. Once this stage is finished, the trial dataset will be declared frozen and exported from the MACRO database for final data review and validation checks by a statistician, who will raise data queries with the trial manager or data manager. Once the trial statistician, data manager, and trial manager are satisfied that all queries have been resolved, the database will be locked. The locked database will be exported to SAS and used for final analysis.

4. Detailed Analysis Plan

4.1 Interim analysis

A group sequential design has been used to allow the Data Monitoring Committee (DMC) to review the primary outcome for evidence of harm, benefit or futility. Two interim analyses will be performed during the study period. The first of these will be after 124 participants have been randomised and reached 7 days post-randomisation and the second after 248 participants have been randomised and reached 7 days post-randomisation. At these points, O'Brien-Fleming stopping rules for harm, benefit or futility will be used to guide the DMC review of the primary outcome data. The stopping rules will be used as a guideline, alongside the other safety data available to the DMC, and used as part of their overall assessment of the trial. They will have overall oversight and can recommend terminating the trial early for these or any other safety concerns. An overall significance level of 5% will be preserved at the end of the trial due to the use of the O'Brien-Fleming stopping boundaries. The interim analysis will be unadjusted.

The interim analysis will be performed by the trial statistician who will be unblinded to the treatment arm and the lead statistician will verify the results. Unblinded results of the interim analysis will be provided to the DMC who will discuss the results. The DMC will report their recommendations to the Trial Steering Committee (TSC), being careful not to reveal any interim trial data that led to their conclusion. The TSC will make the final decision regarding the continuation of the trial. No details on the outcome data should be made available outside the DMC, trial and lead statistician.

There will be no sample size re-estimation during the trial.

4.2 Analysis principles

The population used for efficacy analyses will be a modified intention to treat population including all randomised participants that received a transplant. This is a change from the original proposal because it was deemed illogical to include those participants who have not been transplanted as no outcome data will be available. This will be the primary analysis for the trial. Participants who were not transplanted will be listed with reasons for not being transplanted. The number and percentage of these occurrences

will also be tabulated by trial arm and compared using Fisher's exact test to show any differences between trial arm.

Participants who did not meet all the inclusion criteria and/or met at least one of the exclusion criteria will be considered as randomised in error. Eligible participants who did not receive the randomised treatment, who did not receive at least 1 hour of EVNP (for those randomised to EVNP), or for pairs of kidneys the incorrect order to which they were randomised, will be considered as protocol deviations. Any participants who withdrew their consent to participate in the trial and any withdrawal of participants by their clinician for medical reasons, will be considered as a withdrawn participant. Participants randomised in error, protocol deviations and withdrawn participants will be tabulated separately with reasons.

Participants randomised in error, participants with protocol deviations, those withdrawn or lost to follow up will be included in the modified intention to treat analysis where possible. A withdrawn participant will only be excluded if they withdrew their consent for their data to be used in the trial. For estimation of survival rates, participants lost to follow up will be considered as censored at their last known vital status.

The primary and secondary outcomes will also be analysed per protocol. This analysis will exclude any participant who did not receive a transplant, was randomised in error, was a protocol deviation or was withdrawn from the trial. For both analysis populations (modified intention to treat and per protocol) results will be presented by the treatment to which the participant was randomised. All ratios will be presented as EVNP versus cold storage. A ratio which is greater than 1 indicates that the odds or hazard of the event is greater in the EVNP arm. 95% confidence intervals will be presented with all ratios.

All tests will be two-sided and p-values of less than 0.05 will be considered as statistically significant. P-values will be reported to four decimal places with p-values less than 0.0001 as <0.0001. The statistical package SAS (Cary, NC) will be used to conduct analyses. Multiple comparisons will be performed and this may increase the probability of observing a statistically significant result by chance. No adjustments will be made to account for multiple testing.

Characteristics of all randomised participants will be tabulated by arm of the trial to describe the cohort. This table will also be replicated, excluding those participants who were not transplanted. A CONSORT diagram will be presented to show how participants progressed through the trial. Due to the COVID-19 pandemic, two eligibility cohorts will be presented 1) all eligible patients up till 23 March 2020, when the first COVID-19 lockdown started in the UK and 2) 4 September 2020, when the trial officially closed to recruitment.

4.3 Analysis of primary outcome measures

Any participants who experienced PNF will be excluded from this outcome. The number and proportion of participants who experienced delayed graft function between the two groups will be presented and compared using a logistic regression model adjusting for cold ischaemic time, donor age, left/right kidney and centre (left/right kidney and centre are being included as these were stratification factors within the randomisation list). All of these adjustment factors will be fixed effects in the logistic regression model. For pairs of kidneys, the cold ischaemic time will capture the order in which the kidneys were randomised. The adjusted odds ratio of the treatment effect and associated confidence interval will be presented with the p-value from the likelihood ratio test when including and excluding the treatment term from the model.

Deaths within 7 days post transplant are very rare. Data from the UK Transplant Registry, held by NHS Blood and Transplant shows that over the 5 years between 1 January 2013 and 31 December 2017, of all 3,880 DCD single kidney transplants performed in the UK, there was only 3 deaths in the first 7 days post transplant (0.08%). Therefore, it was assessed that a death as a competing risk analysis was not required here.

4.4 Analysis of secondary outcome measures

- *Primary non-function:*
  Primary non-function (PNF) defined as the permanent lack of allograft function from the time of transplantation. This will include graft losses due to irreversible rejection and vascular thrombosis. The causes of graft failure will be tabulated by treatment arm, after being reviewed (blinded to treatment arm) by the Chief Investigator and Trial Manager, to ensure the correct classifications. The number and proportion of participants who experienced PNF between the two groups will be presented and compared using an exact logistic regression model adjusting for cold ischaemic time, donor age, left/right kidney and centre (all as fixed effects). An exact logistic regression model was chosen because it is anticipated that PNF will occur infrequently (<5%). If the exact logistic regression model is computationally infeasible, then cold ischaemic time will be rounded to hours to see if this alleviates the problems. Failing this, standard logistic regression will be used. For pairs of kidneys, the cold ischaemic time will capture the order in which the kidneys were randomised. The adjusted odds ratio of the treatment effect and associated confidence interval will be presented with the p-value from the likelihood ratio test when including and excluding the treatment term from the model.
- *Duration of DGF in days:*
  The duration of DGF will be calculated as the number of days between revascularisation (clamp release) and when the post-transplant dialysis finished. If the dates are the same, the duration will be set to 0. This outcome will only include those participants who have experienced DGF (and did not experience PNF) and deaths will be censored. The unadjusted median (and interquartile range) duration of DGF will be obtained from the Kaplan Meier estimate and will be presented by trial arm, for all those who experienced DGF. Difference in duration of DGF will be assessed through the use of a Cox Proportional Hazards Regression model, adjusting for cold ischaemic time, donor age, left/right kidney indicator and centre, which will all be treated as fixed effects. The hazard ratio of the treatment variable will be presented with 95% confidence interval and the p-value from the likelihood ratio test when comparing models with and without the treatment effect.
- *Functional DGF:*
  Functional DGF (fDGF) will be defined as <10% fall in serum creatinine for 3 consecutive days in the first week post-transplant, hence will be captured on day 3 to day 7 post-transplant. This outcome will exclude any participants who have experienced DGF or PNF as it is meaningless to include them. The number and proportion of participants who experienced fDGF between the two treatment arms will be presented and compared using a logistic regression model adjusting for cold ischaemic time, donor age, left/right kidney and centre (all fixed effects). For pairs of kidneys, the cold ischaemic time will capture the order in which the kidneys were randomised. The adjusted odds ratio of the treatment effect and associated confidence interval will be presented with the p-value from the likelihood ratio test when including and excluding the treatment term from the model.
- *Creatinine reduction ratio day 2*:
  The creatinine reduction ratio on day 2 (CRR2) will be calculated as (creatinine day 1 – creatinine day 2)/creatinine day 1). This outcome will exclude any participants who

have experienced DGF or PNF as it is meaningless to include them. The unadjusted mean and standard deviation CRR2 will be presented and compared using a normal linear regression model adjusting for cold ischaemic time, donor age, left/right kidney and centre (all fixed effects). The adjusted mean treatment difference, from the normal linear regression model, will be presented with 95% confidence interval. The p-value from the likelihood ratio test comparing models with and without the treatment term in the model will also be presented.

- *Creatinine reduction ratio day 5:*
  The creatinine reduction ratio on day 5 (CRR5) will be calculated as (creatinine day 1 – creatinine day 5)/creatinine day 1). This outcome will exclude any participants who have experienced DGF or PNF as it is meaningless to include them. The unadjusted mean and standard deviation CRR5 will be presented and compared using a normal linear regression model adjusting for cold ischaemic time, donor age, left/right kidney and centre (all fixed effects). The adjusted mean treatment difference, from the normal linear regression model, will be presented with 95% confidence interval. The p-value from the likelihood ratio test comparing models with and without the treatment term in the model will also be presented.
- *Length of hospital stay in days:*
  This will be calculated as the time from hospital admission to discharge and deaths will be censored if the participant experienced death before discharge, as will participants who are lost to follow up. The unadjusted median (and interquartile range) duration of hospital stay will be obtained from the Kaplan Meier estimate and will be presented by trial arm. Difference in duration of hospital stay will be assessed through the use of a Cox Proportional Hazards Regression model, adjusting for cold ischaemic time, donor age, left/right kidney indicator and centre, which will all be treated as fixed effects. The hazard ratio of the treatment variable will be presented with 95% confidence interval and the p-value from the likelihood ratio test when comparing models with and without the treatment effect. A competing risks analysis was considered unnecessary for this secondary outcome: data from the UK Transplant Registry, held by NHS Blood and Transplant shows that over the 5 years between 1 January 2013 and 31 December 2017, of all 3,880 DCD single kidney transplants performed in the UK, there was only 9 deaths in the first 14 days post transplant (0.2%) and 12 deaths within the first 21 days post transplant (0.3%).
- *Biopsy-proven acute rejection rate:*
  The number of biopsy-proven rejection episodes per participant will be summarised as an unadjusted mean and standard deviation by treatment arm. The number of episodes per participant will be compared between treatment arms using a negative binomial model adjusted for cold ischaemic time, donor age, left/right kidney and centre (all as fixed effects). The adjusted rate ratio from this model will be presented with the 95% confidence interval. The p-value from the likelihood ratio test, with and without the treatment effect will be presented.

- *Serum creatinine and eGFR at 1, 3, 6 and 12 months:*
  eGFR will be calculated using the MDRD formula[1], which utilises the recipients' sex, age, ethnicity and serum creatinine. This outcome will exclude any participants who have experienced DGF or PNF as it is meaningless to include them. Unadjusted mean and standard deviation of serum creatinine, and separately eGFR, will be presented at 1, 3, 6 and 12 months post-transplant for each trial arm. To assess if there is a difference in serum creatine (and separately eGFR) between the two arms

[1] Levey, A, Greene, T, Kusek, J, and Beck, G. A simplified equation to predict glomerular filtration rate from serum creatinine. J Am Soc Nephrol. 2000; 11: A0828 ((abstr).)

over all time points, a normal linear regression model will be fitted adjusting for cold ischaemic time, donor age, left/right kidney, centre, time of measurement and a time by treatment interaction term (all as fixed effects). A random effect will also be included to allow for multiple measures per recipient. If the likelihood ratio test shows that the interaction term is significant then the adjusted mean treatment by time difference and 95% confidence interval will be presented, alongside the p-value from the likelihood ratio test. If the likelihood ratio test shows that the interaction term is not significant then the individual adjusted mean treatment and time terms will be presented with their 95% confidence intervals and associated p-values from the likelihood ratio test. Despite whether the interaction term is significant or not, the adjusted means and their standard errors for each time point will be plotted to visually see the effects of serum creatine (and separately eGFR) over time. These adjusted means and standard errors will come from the model from which the covariates are presented.

- *Patient survival (time from transplant to death):*
  This will be calculated in days and participants who are lost to follow up will be censored. A Kaplan-Meier survival plot will be presented to show the unadjusted probability of patient death after 12 months by treatment arm. Difference in patient survival will be assessed using a Cox Proportional Hazards Regression model, adjusting for cold ischaemic time, donor age, left/right kidney indicator and centre, which will all be treated as fixed effects. The hazard ratio of the treatment variable will be presented with 95% confidence interval and the p-value from the likelihood ratio test when comparing models with and without the treatment effect.
- *Allograft survival (time from transplant to graft loss or return to dialysis):*
  Time to graft loss or return to dialysis in days will be modelled. Deaths will be censored, if the participant experienced death before the graft failure, as will participants who are lost to follow up. A Kaplan-Meier survival plot will be presented to show the unadjusted probability of graft failure after 12 months by arm. Difference in graft survival will be assessed using a Cox Proportional Hazards Regression model, adjusting for cold ischaemic time, donor age, left/right kidney indicator and centre, which will all be treated as fixed effects. The hazard ratio of the treatment variable will be presented with 95% confidence interval and the p-value from the likelihood ratio test when comparing models with and without the treatment effect.

4.5 Other outcome measures

Safety outcomes

The complications which will be examined are:

6. Acute rejection
7. Renal artery thrombosis
8. Renal vein thrombosis
9. The complications of renal transplant biopsy
10. Number of hospital admissions for any recognised complication of renal transplantation and immunosuppression

Total number of incidences of each complication will be tabulated arm. The number of each complication per participant will be summarised as an unadjusted mean and standard deviation by treatment arm. The number of each complication per participant will be compared between treatment arms using a Negative Binomial model adjusted for cold ischaemic time, donor age, left/right kidney and centre (all as fixed effects). The adjusted rate ratio (and 95% confidence interval) will be presented with the p-value from the likelihood ratio test with and without the treatment effect.

Other outcomes

EVNP assessment score

For those participants who received EVNP, the score will be tabulated with numbers and percentages. To assess for associations between DGF and the EVNP assessment score a logistic regression model will be fitted, adjusting for cold ischaemic time, donor age, left/right kidney and centre (all as fixed effects). EVNP assessment score will be fitted as a categorical variable. The adjusted odds ratio for the different levels of the EVNP assessment score will be presented with the 95% confidence interval and the p-value from the likelihood ratio test when including and excluding the EVNP assessment score term from the model.
Other characteristics will be assessed to see if they are important in predicting DGF after accounting for the EVNP assessment score and the other adjustment factors. The following characteristics will be assessed for inclusion within the logistic regression model: donor characteristics: age, sex, ethnicity, BMI, past diabetes and past hypertension, and recipient characteristics: age, sex, ethnicity, diabetes status, pre-transplant serum creatinine, pre-transplant eGFR, dialysis status. A backward stepwise selection method, using the likelihood ratio test with a 5% significant level will be used to determine which factors remain in the model. The final model will be presented with the odds ratios and associated confidence intervals, and the p-value from the likelihood ratio test when omitting the term from the model.

Ancillary Studies
Details of the analysis for the ancillary studies will be specified in a separate analysis plan(s).

4.6 Sub-Group analyses

None.

4.7 Sensitivity analyses

To explore if missing values have an undue impact on the primary outcome result, a sensitivity analysis using multiple imputation will be performed if delayed graft function is missing in more than 2% of the participants included in the modified intention to treat analysis. Variables will be included in the imputation model if they have a completeness of ≥65% and these variables are donor age, donor height, donor weight, donor retrieval creatinine, donor sex, donor blood group, donor ethnicity, donor cause of death, donor past history of hypertension, recipient age, recipient waiting time, recipient sex, recipient blood group, recipient ethnicity, recipient dialysis status prior to transplant, recipient diabetes status, whether the recipient was highly sensitised (calculated reaction frequency of ≥85%), left/right kidney, transplanting centre, cold ischaemic time and HLA mismatch between donor and recipient. Forty imputations will be used and the average across the imputations will be calculated. The model specified in section 4.3 will be utilised. If the proportion of participants with a missing delayed graft function outcome is less than or equal to 2% then this sensitivity analysis will not be performed.

None of the secondary nor other outcomes will be replicated in a sensitivity analysis.

4.8 Procedures for handling Missing Data, Protocol Deviations, patients randomised in error and Losses to Follow-up

Any missing primary and secondary outcome data will be summarised. Primary and secondary outcome measures will not be imputed and these will be treated as missing data and excluded from the relevant analyses. Imputation will only be conducted in the sensitivity analysis, as described in section 4.7. If outcome data is missing for more than 25% of participants, outcomes will not be reported.

Protocol deviations, participants randomised in error, lost to follow-up or withdrawn have been described in section 4.2, including how these will be handled in the analysis.

5. Data Analysis Tables to be Completed

5.1 Screening, Recruitment and Follow-up tables

5.1.1 Recruitment by Centre

Table 1 Recruitment by Centre

Site	**Number eligible up to 23 Mar 2020***	**Number eligible up to 4 Sep 2020***	**Number consented**	% consented from eligible up to 23 Mar 2020	% consented from eligible up to 4 Sep 2020	**Number randomised**	% rand from eligible up to 23 Mar 2020	% rand from eligible up to 4 Sep 2020
Cambridge								
Edinburgh								
Guy’s								
Newcastle								
Royal London **								
Glasgow**								

*Extracted from the UK Transplant Registry, held by NHS Blood and Transplant
** These two sites never opened to recruitment

5.1.2 Patients withdrawn

Table 2 Participants withdrawn

Note: this table excludes any participants who were withdrawn because they were not transplanted

Participant ID	Randomised treatment	Reason for withdrawal

5.1.3 Patients randomised in error

Table 3 Participants randomised in error

Note: this table excludes all participants who not transplanted

Participant ID	Randomised treatment	Detail of error

5.1.4 Protocol Deviations

**Table 4**	**Protocol deviations**<br>Note: this table excludes all participants who not transplanted	
Participant ID	Randomised treatment	Detail of protocol deviation

**Table 5**	**Participants not transplanted**			
**Summary of reasons**				
Participant ID	Randomised treatment	Reason for not being transplanted		
**Totals**				
	**CS alone**	**EVNP**	**Total**	**P-value[1]**
Not transplanted				
Transplanted				
[1] Fishers exact test				

5.2 Baseline Characteristics tables

**Table 6**	**Characteristics- data are N/Total N (%) for categorical variables and mean (IQR) for continuous variables**					
**Characteristic**	**Randomised treatment**				**Total (n=)**	
	**CS alone (n=)**		**EVNP (n=)**			
**Donor**						
Age						
Male						
Ethnicity<br>White<br>Black<br>Asian<br>Other						
BMI						

Past diabetes						
Past hypertension						
**Recipient**						
Age						
Male						
Ethnicity						
White<br>Black<br>Asian<br>Other						
Diabetic						
Pre transplant SCr						
Pre transplant eGFR						
On dialysis						
Type of dialysis						
Haemodialysis<br>Peritoneal dialysis						
**Transplant**						
Left kidney transplant						
Cold ischaemic time						
Duration of EVNP						
SCr= Serum creatinine. eGFR= estimated glomerular filtration rate.<br>Summary of missing data:						

5.3 Primary Outcome table

**Table 7**	**Primary outcome**			
	**Modified intention-to-treat**		**Per-protocol**	
**Outcome**	**CS alone (n=)**	**EVNP (n=)**	**CS alone (n=)**	**EVNP (n=)**
Delayed graft function				
N/Total N (%)				
OR (95% CI)[1]				
p-value[2]				

[1] Logistic regression model adjusted for cold ischaemic time, donor age, left/right kidney indicator and centre.
[2] p-value from the likelihood ratio test when including and excluding the treatment term from the model.

5.4 Secondary Outcome tables

**Table 8 Secondary outcomes**				
	**Modified intention-to-treat**		**Per-protocol**	
**Outcome**	**CS alone (n=)**	**EVNP (n=)**	**CS alone (n=)**	**EVNP (n=)**
Primary non-function				
N/Total N (%)				
OR (95% CI)[1]				
p-value[1]				
Cause of graft failure N (%)				
Hyper acute rejection				
Rejection while taking immunosuppressive drugs				
Rejection after stopping all immunosuppressive drugs				
Vascular (arterial or venous) thrombosis				
Cortical necrosis				
Not available				
Duration of DGF (days)				
Total N				
Median (IQR)[2,3]				
HR (95% CI)[4]				
p-value[4]				
Functional DGF				
N/Total N (%)				
OR (95% CI)[1]				
p-value[1]				
Creatinine reduction ratio day 2				
Total N				
Mean (SD)[2]				
Mean difference (95% CI)[5]				
p-value[5]				
Creatinine reduction ratio day 5				
Total N				
Mean (SD)[2]				
Mean difference (95% CI)[5]				
p-value[5]				
Length of hospital stay (days)				
Total N				
Median (IQR)[4]				
HR (95% CI)[4]				
p-value[4]				
Biopsy-proven acute rejection rates				
Total N				
Mean (SD) per participant [2]				
Rate ratio (95%CI)[6]				
p-value[6]				
Serum creatinine				

**Table 8**	**Secondary outcomes**				
		**Modified intention-to-treat**		**Per-protocol**	
**Outcome**		**CS alone (n=)**	**EVNP (n=)**	**CS alone (n=)**	**EVNP (n=)**
at 1 month	Total N				
	Mean (SD)[2]				
at 3 months	Total N				
	Mean (SD)[2]				
at 6 months	Total N				
	Mean (SD)[2]				
at 12 months	Total N				
	Mean (SD)[2]				
Covariates[5]					
p-value[5]					
eGFR					
at 1 month	Total N				
	Mean (SD)[2]				
at 3 months	Total N				
	Mean (SD)[2]				
at 6 months	Total N				
	Mean (SD)[2]				
at 12 months	Total N				
	Mean (SD)[2]				
Covariates[5]					
p-value[5]					
Patient survival					
Total N					
Probability of patient death after 12 months[2]					
HR (95% CI)[4]					
p-value[4]					
Graft survival					
Total N					
Probability of graft failure after 12 months[2]					
HR (95% CI)[4]					
p-value[4]					

[1] Logistic regression model
[2] Unadjusted
[3] Kaplan Meier estimate
[4] Cox proportional hazards regression model
[5] Normal linear regression model
[6] Negative binomial model

All ratios, mean differences and p-values have been adjusted for cold ischaemic time, donor age, left/right kidney indicator and centre. All p-values are from the likelihood ratio test when including and excluding the treatment term from the model.

5.5 Safety data summary tables

**Table 9**	**Safety outcomes- Modified intention to treat**		
**Complication**	**CS alone (n=)**	**EVNP (n=)**	**Total**
Acute Rejection			
Total number			
Mean (SD) per participant[1]			
Rate ratio (95% CI)			
p-value			
Renal artery thrombosis			
Total number			
Mean (SD) per participant[1]			
Rate ratio (95% CI)			
p-value			
Renal vein thrombosis			
Total number			
Mean (SD) per participant[1]			
Rate ratio (95% CI)			
p-value			
The complications of renal transplant biopsy			
Total number			
Mean (SD) per participant[1]			
Rate ratio (95% CI)			
p-value			
Hospital admission for any recognised complication of renal transplantation and immunosuppression			
Total number			
Mean (SD) per participant[1]			
Rate ratio (95% CI)			
p-value			

[1] Unadjusted
All ratios from a negative binomial model adjusted for cold ischaemic time, donor age, left/right kidney and centre. All p-values are adjusted and from likelihood ratio test with and without the treatment effect.

**Table 10**	**Other outcomes- EVNP Assessment Score (n=)**			
EVNP assessment score	N	%	Adjusted odds ratio (95% CI)[1]	p-value[2]
1				
2				
3				
4				
5				
Characteristic A				
Characteristic B				
Characteristic C				
….				

[1] To assess for associations between DGF and the EVNP assessment score, using a logistic regression model, adjusted for cold ischaemic time, donor age, left/right kidney,

centre and any other donor or recipient characteristic found to be significant at the 5% level.<br>[2] P-value from the likelihood ratio test when including and excluding the term from the model

5.6 Sub-group analysis table

None

5.7 Missing Data and Sensitivity analysis tables

**Table 11**	**Sensitivity analysis**			
	**Modified intention-to-treat**		**Per-protocol**	
**Outcome**	**CS alone (n=)**	**EVNP (n=)**	**CS alone (n=)**	**EVNP (n=)**
**Effect of missing data (after conducting multiple imputation)**				
Delayed graft function				
N (%)				
OR (95% CI)[1]				
p-value[2]				
[1] Logistic regression model adjusted for cold ischaemic time, donor age, left/right kidney indicator and centre.<br>[2] p-value from the likelihood ratio test when including and excluding the treatment term from the model.				

**Table 12** **Missing data table n (% of column total)**<br>Note: this table excludes all participants who not transplanted					
	**CS alone (n=)**		**EVNP (n=)**		**Total (n=)**
Missing primary outcome					
Inferred primary outcome					
Missing PNF					
Missing duration of DGF					
Missing fDGF					
Missing CRR2					
Missing CRR5					
Missing length of hospital stay					
Missing biopsy proven acute rejection episodes					
Missing serum creatinine					
At 1 month					
At 3 months					
At 6 months					
At 12 months					
Missing eGFR					
At 1 month					
At 3 months					

At 6 months					
At 12 months					
Missing 12 month patient survival					
Missing 12 month graft survival					

5.8 Figures

Figure 1: Consort diagram

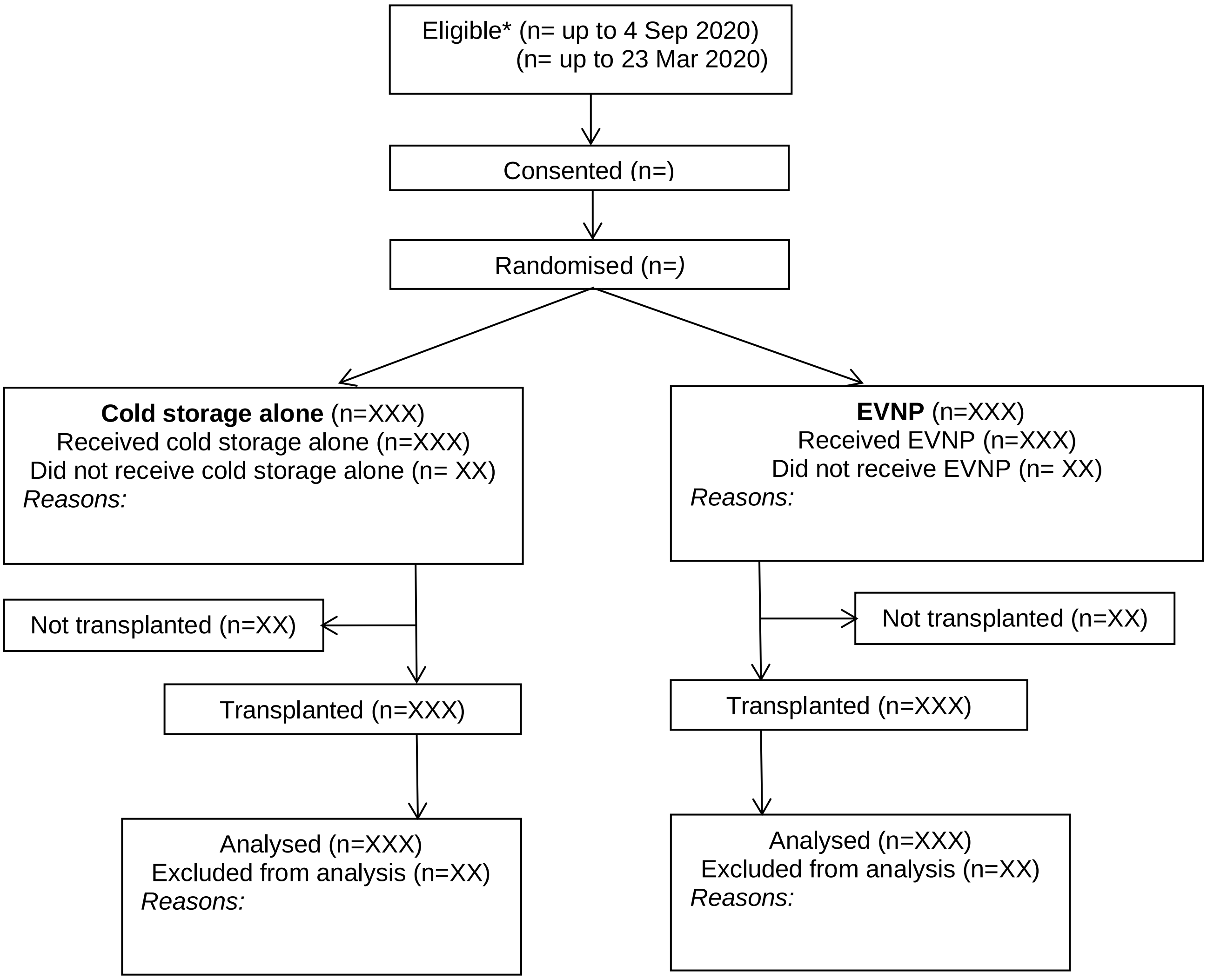

*Extracted from the UK Transplant Registry, held by NHS Blood and Transplant

Figure 2: Adjusted mean and standard errors for 1, 3, 6 and 12-month serum creatinine

Figure 3: Adjusted mean and standard errors for 1, 3, 6 and 12-month eGFR

Figure 4: Kaplan Meier Survival Plot for 12-month patient survival

Figure 5: Kaplan Meier Survival Plot for 12-month graft survival

6.0 Statistical Analysis Plan Amendments

Revision History:

Version	Author	Date	Reason for revision
1.1	Laura Pankhurst	03/03/2021	Align with version 1.4 of the protocol and a few minor updates in section 4.4
1.2	Laura Smith	16/08/2021	Clarifying that the PNF cases should be excluded from several outcomes, additional eligibility cohort due to COVID-19 pandemic and a few other minor updates/clarifications

Appendix 2

Amendment history

List details of all protocol amendments here whenever a new version of the protocol is produced. Protocol amendments must be submitted to the Sponsor for approval prior to submission to the REC committee.

**Amendment No.**	**Protocol version no.**	**Date issued**	**Author(s) of changes**	**Details of changes made**
1	1.1	4th Nov 2015	S Hosgood	Amendment of the rate of risk
2	1.2	11th August 2016	S Hosgood	Addition of supplementary study and minor amendments to the protocol
3	1.3	10th May 2017	S Hosgood	**Addition of exclusion criteria**<br>1: 1: If a kidney is randomized as a single and the other paired kidney is accepted by the centre at a later time, the second kidney will not be included in the trial.<br>2: If the donor undergoes normothermic regional perfusion prior to retrieval of the organs.<br><br>**Changes to the immunosuppression protocol**<br>'Campath may be used as an induction therapy as an alternative to Basiliximab'<br><br>**Statistical section**<br>More information has been added to the statistics section. This is to clarify when the interim analyses will be carried out and procedures that will take place following this.<br><br>**Minor Amendments**<br>1: Valerie Hopkins is the trial data manager<br>2: The calculation for the creatinine reduction ratio on day 5 has been corrected. . Creatinine reduction ratio day 5 (CRR 5 = Day 1 creatinine - creatinine day 5/ pre-transplant creatinine).<br>3: Section 7.2 'Expected serious adverse events recognized to be caused by EVNP should read'

				There is a small risk of damage to the kidney vessels during preparation for EVNP. This risk is minimal (<1%).<br><br>4: Section 8.6.1 ‘Analysis of primary and secondary outcomes’ Serum creatinine and eGFR will be measured at 1, 3, 6 and 12 months: longitudinal changes in serum creatinine and eGFR will be separately assessed at 1, 3, 6 and 12 months post-transplant and mean values compared using two-way ANOVA with repeated measures for patients in each arm of the trial. The protocol previously stated 3, 6 and 12 months only.
4	1.4	5th May 2019	S Hosgood	**Major**<br>The addition of 2 more centres. Queen Elizabeth Hospital, Glasgow and The Royal London<br><br>Kidney recruited by The Royal London will undergo EVNP at Guy’s & St Thomas’s<br><br>**Minor**<br>Siobhan Martin has replaced Valerie Hopkins as the data manager.<br>Grant Stewart has replaced Paul Hayes in the Trial Steering Committee.<br><br>The following statistical sections have been updated<br>8.4, 8.5, 8.6, 8.6.1, 8.6.2
