# Supplementary material for: Normothermic machine perfusion versus static cold storage in donation after circulatory death kidney transplantation: a randomized controlled trial
Source: Nat Med. 2023 May 25;29(6):1511–9. doi: 10.1038/s41591-023-02376-7 (PMC10287561; doi:10.1038/s41591-023-02376-7)
Supplement: Supplementary file 2 — Reporting Summary [file 41591_2023_2376_MOESM2_ESM.pdf]

nature portfolio

Corresponding author(s): Dr Sarah Hosgood

Last updated by author(s): Apr 20, 2023

Reporting Summary

Nature Portfolio wishes to improve the reproducibility of the work that we publish. This form provides structure for consistency and transparency in reporting. For further information on Nature Portfolio policies, see our Editorial Policies and the Editorial Policy Checklist.

Statistics

For all statistical analyses, confirm that the following items are present in the figure legend, table legend, main text, or Methods section.

n/a	Confirmed	
☐	☒	The exact sample size (*n*) for each experimental group/condition, given as a discrete number and unit of measurement
☐	☒	A statement on whether measurements were taken from distinct samples or whether the same sample was measured repeatedly
☐	☒	The statistical test(s) used AND whether they are one- or two-sided<br>*Only common tests should be described solely by name; describe more complex techniques in the Methods section.*
☐	☒	A description of all covariates tested
☐	☒	A description of any assumptions or corrections, such as tests of normality and adjustment for multiple comparisons
☐	☒	A full description of the statistical parameters including central tendency (e.g. means) or other basic estimates (e.g. regression coefficient) AND variation (e.g. standard deviation) or associated estimates of uncertainty (e.g. confidence intervals)
☐	☒	For null hypothesis testing, the test statistic (e.g. *F*, *t*, *r*) with confidence intervals, effect sizes, degrees of freedom and *P* value noted<br>*Give P values as exact values whenever suitable.*
☒	☐	For Bayesian analysis, information on the choice of priors and Markov chain Monte Carlo settings
☒	☐	For hierarchical and complex designs, identification of the appropriate level for tests and full reporting of outcomes
☒	☐	Estimates of effect sizes (e.g. Cohen's *d*, Pearson's *r*), indicating how they were calculated

Our web collection on statistics for biologists contains articles on many of the points above.

Software and code

Policy information about availability of computer code

Data collection	Data was collected prospectively at each of the trial centres and by NHSBT using Macro database version 4.9.
Data analysis	SAS Enterprise Guide (version 7.15) with SAS 9.4 was used to conduct all analyses. The randomisation list was created by the trial statistician in SAS Enterprise Guide (version 5.1) with SAS 9.4.

For manuscripts utilizing custom algorithms or software that are central to the research but not yet described in published literature, software must be made available to editors and reviewers. We strongly encourage code deposition in a community repository (e.g. GitHub). See the Nature Portfolio guidelines for submitting code & software for further information.

Data

Policy information about availability of data

All manuscripts must include a data availability statement. This statement should provide the following information, where applicable:

- Accession codes, unique identifiers, or web links for publicly available datasets
- A description of any restrictions on data availability
- For clinical datasets or third party data, please ensure that the statement adheres to our policy

Data from the trial are stored in an online secure database hosted by NHSBT Clinical Trials Unit. The protocol, consent form, statistical analysis plan, definition and derivation of clinical characteristics and outcomes, training materials, regulatory documents, and other relevant study materials are available online and have been published elsewhere. The datasets generated during analysis will be available upon request from the NHSBT Clinical Trials Unit after deidentification (text, tables,

figures and appendices) 9 months after publication and ending 5 years following article publication. Data will be shared with investigators whose use of the data has been assessed and approved by an NHSBT review committee as a methodologically sound proposal. NHSBT's clinical trials unit can be contacted at CTU@nhsbt.nhs.uk. The CTU will be able to provide a copy of our data sharing policy and arrange a data use agreement which will need to be signed. All data use agreements will be in line with the consent given by participants upon agreeing to take part in the trial.

Human research participants

Policy information about studies involving human research participants and Sex and Gender in Research.

Reporting on sex and gender

Sex or gender have been stated in the study. The study includes patients of both sex. Analysis does not include sex or gender related differences.

Population characteristics

Patients with end stage renal failure requiring a kidney transplant were included in the study. The median age was 59 years (50-66).

Recruitment

Eligible patients enrolled on the transplant waiting list which were allocated a suitably matched kidney were enrolled at four UK transplant centres. Recipients 18 years of age or older with end-stage renal failure requiring their first or second kidney transplant who received a kidney from Maastricht category III or IV DCD donors 18 years of age or older were included in the trial. Patients were randomly assigned in a 1:1 ratio to 1h NMP or SCS. The randomisation list was created by the trial statistician in SAS Enterprise Guide (version 5.1) with SAS 9.4, stratified by centre and using randomly permuted blocks of fixed size 2 and 4 for single and pairs of kidneys respectively. In cases where paired kidneys from the same donor were transplanted in the same centre, the randomisation was stratified by kidney (right or left) so that one kidney was randomly allocated to each treatment and in which order they should be transplanted. The randomisation process was facilitated using an Interactive Web Response System. After the assignment of treatment arms, no-one in the trial was blinded to the treatment allocation.

Ethics oversight

This investigator-led randomised controlled open label trial was approved by the UK National Research Ethics Service and local institutional review boards (REC 15/EE/0356)

Note that full information on the approval of the study protocol must also be provided in the manuscript.

Field-specific reporting

Please select the one below that is the best fit for your research. If you are not sure, read the appropriate sections before making your selection.

☒ Life sciences ☐ Behavioural & social sciences ☐ Ecological, evolutionary & environmental sciences

For a reference copy of the document with all sections, see nature.com/documents/nr-reporting-summary-flat.pdf

Life sciences study design

All studies must disclose on these points even when the disclosure is negative.

Sample size

Historical data spanning a 5-year period for three participating centres showed that the overall rate of DGF in DCD kidney transplants was 50%. This was used as our baseline rate. In a pilot series of kidney transplants from extended criteria donors (ECD), 18 kidneys undergoing SCS followed by 1h of NMP were compared to a historical control group of 47 ECD transplants after SCS alone. The DGF rates were 1/18 (6%) in the NMP group compared to 17/47 (36%) in the SCS group. Using a fixed sample size study, with interim analyses after 124 and 248 participants had been enrolled and reached 7 days post-transplant, a total of 370 patients receiving a DCD kidney were required to detect a 30% relative reduction in DGF (from 50% to 35%) with a power of 80%, a statistical significance of $\alpha = 0.05$ and 1-1 allocation. To allow for a study withdrawal rate of 7.5%, a maximum of 400 patients were needed for recruitment.

Data exclusions

The primary outcome was analysed using an adjusted logistic regression model and excluded participants who experienced PNF. The data for this outcome were complete and therefore it was not necessary to undertake any of the methods proposed in the statistical analysis plan for assessing the impact of these missing data.

Replication

Quality control of data entered and data cleaning were performed by the trial data manager. For the final analysis, the primary outcome was independently replicated by the Lead Statistician for the trial for both the modified ITT and per protocol cohorts. The post-hoc subgroup analysis was also independently replicated. The final analysis was replicated once at the time of final analysis. The interim analyses were also independently replicated (once for the first interim and once for the second). All attempts at replication were successful.

Randomization

Patients were randomly assigned in a 1:1 ratio to 1h NMP or SCS. The randomisation list was created by the trial statistician in SAS Enterprise Guide (version 5.1) with SAS 9.4, stratified by centre and using randomly permuted blocks of fixed size 2 and 4 for single and pairs of kidneys respectively. In cases where paired kidneys from the same donor were transplanted in the same centre, the randomisation was stratified by kidney (right or left) so that one kidney was randomly allocated to each treatment and in which order they should be transplanted. The randomisation process was facilitated using an Interactive Web Response System.

Blinding

No-one in the trial was blinded to the treatment allocation. For safety and logistical reasons the transplanting surgeon needed to know the

Blinding	group allocation of kidney

Reporting for specific materials, systems and methods

We require information from authors about some types of materials, experimental systems and methods used in many studies. Here, indicate whether each material, system or method listed is relevant to your study. If you are not sure if a list item applies to your research, read the appropriate section before selecting a response.

Materials & experimental systems

n/a	Involved in the study
☒	☐ Antibodies
☒	☐ Eukaryotic cell lines
☒	☐ Palaeontology and archaeology
☒	☐ Animals and other organisms
☐	☒ Clinical data
☒	☐ Dual use research of concern

Methods

n/a	Involved in the study
☒	☐ ChIP-seq
☒	☐ Flow cytometry
☒	☐ MRI-based neuroimaging

Clinical data

Policy information about clinical studies

All manuscripts should comply with the ICMJE guidelines for publication of clinical research and a completed CONSORT checklist must be included with all submissions.

Clinical trial registration	ISRCTN15821205
Study protocol	BMJ OPEN 2017 Jan 23;7 (1) e012237
Data collection	Data was collected at each of the trial centres (Addenbrooke's, Guy's & St Thomas's, Freeman and Edinburgh Royal Infirmary hospitals) and entered onto a Macro database. Data was also collected from the NHSBT National Transplant Registry. Data was collected from February 2016 to September 2021.
Outcomes	The primary outcome was delayed graft function, defined as the requirement for dialysis within the first 7 days post transplant. It was analysed using an adjusted logistic regression model and excluded participants who experienced PNF. The data for this outcome were complete and therefore it was not necessary to undertake any of the methods proposed in the statistical analysis plan for assessing the impact of these missing data.<br><br>Secondary and safety outcome measures were analysed using logistic regression model (PNF, functional DGF), Cox proportional hazards model (duration of DGF, length of hospital stay, allograft and patient survival), normal linear regression model (CRR at day 2 and 5, serum creatinine and eGFR at 1, 3, 6 and 12 months) and negative binomial model (biopsy-proven acute rejection and safety outcomes). Missing secondary outcome measures were not imputed and excluded from the relevant analyses. Full details can be found in the statistical analysis plan. To ensure model assumptions were met, residual plots were examined.<br>The primary outcome was analysed using an adjusted logistic regression model and excluded participants who experienced PNF. The data for this outcome were complete and therefore it was not necessary to undertake any of the methods proposed in the statistical analysis plan for assessing the impact of these missing data.
